# Potent hemithioindigo-based antimitotics photocontrol the microtubule cytoskeleton in cellulo

**DOI:** 10.3762/bjoc.16.14

**Published:** 2020-01-27

**Authors:** Alexander Sailer, Franziska Ermer, Yvonne Kraus, Rebekkah Bingham, Ferdinand H Lutter, Julia Ahlfeld, Oliver Thorn-Seshold

**Affiliations:** 1 Department of Pharmacy, Ludwig Maximilian University of Munich, Butenandtstraße 5-13, Munich 81377, Germany

**Keywords:** antimitotics, cytoskeleton, hemithioindigo, photopharmacology, photoswitch

## Abstract

**Background:** Hemithioindigo is a promising molecular photoswitch that has only recently been applied as a photoswitchable pharmacophore for control over bioactivity in cellulo. Uniquely, in contrast to other photoswitches that have been applied to biology, the pseudosymmetric hemithioindigo scaffold has allowed the creation of both dark-active and lit-active photopharmaceuticals for the same binding site by a priori design. However, the potency of previous hemithioindigo photopharmaceuticals has not been optimal for their translation to other biological models.

**Results:** Inspired by the structure of tubulin-inhibiting indanones, we created hemithioindigo-based indanone-like tubulin inhibitors (**HITubs**) and optimised their cellular potency as antimitotic photopharmaceuticals. These **HITubs** feature reliable and robust visible-light photoswitching and high fatigue resistance. The use of the hemithioindigo scaffold also permitted us to employ a *para*-hydroxyhemistilbene motif, a structural feature which is denied to most azobenzenes due to the negligibly short lifetimes of their metastable *Z*-isomers, which proved crucial to enhancing the potency and photoswitchability. The **HITubs** were ten times more potent than previously reported hemithioindigo photopharmaceutical antimitotics in a series of cell-free and cellular assays, and allowed robust photocontrol over tubulin polymerisation, microtubule (MT) network structure, cell cycle, and cell survival.

**Conclusions: HITubs** represent a powerful addition to the growing toolbox of photopharmaceutical reagents for MT cytoskeleton research. Additionally, as the hemithioindigo scaffold allows photoswitchable bioactivity for substituent patterns inaccessible to the majority of current photopharmaceuticals, wider adoption of the hemithioindigo scaffold may significantly expand the scope of cellular and in vivo targets addressable by photopharmacology.

## Introduction

The cytoskeletal scaffolding protein tubulin, a heterodimer consisting of α and β subunits, each of various isotypes, reversibly assembles into giant non-covalent polymeric microtubules (MTs), which play a pivotal role as a dynamic scaffold for a multitude of cellular processes. These include mechanostasis, the completion of mitosis, cell motility, and cargo trafficking in all cell types, as well as cell-type-specific roles, such as polarization, cargo sorting, and trafficking in neurons; the regulation and functioning of these processes is still not satisfactorily understood [1–4]. The MT cytoskeleton is a finely tuned complex system that is highly conserved through evolution. Direct genetic modifications of tubulin that affect its functions risk causing a diversity of effects, due to its many survival-critical roles, as well as non-functionality of the modified tubulin product. For example, knockout approaches have only been described for single isoforms of α/β-tubulin, and these cannot deliver the dynamic reversibility and effect-specificity that is required for understanding MT biology; and optogenetic modifications of tubulin have never succeeded. Instead, studies of the roles of MTs in these processes overwhelmingly rely on small molecule tubulin inhibitors [1].

Due to the non-invasiveness and high spatiotemporal precision with which optical stimulation can be applied, photopharmacology has drawn great interest for studies of crucial biological processes in a range of fields, from neuroscience [5–6] and G-protein-coupled receptor (GPCR) function [7–8] to antibiotic research [9]. Particularly in the context of MT biology, photopharmacology is an attractive development beyond classical small molecule inhibitors; since the spatiotemporal complexity inherent to the diversity of tubulin-dependent cellular processes may finally yield to studies that can leverage high-spatiotemporal-specificity optical control to deliver cell-specific, time-reversible modulation of native cytoskeleton function.

**Figure 1:**
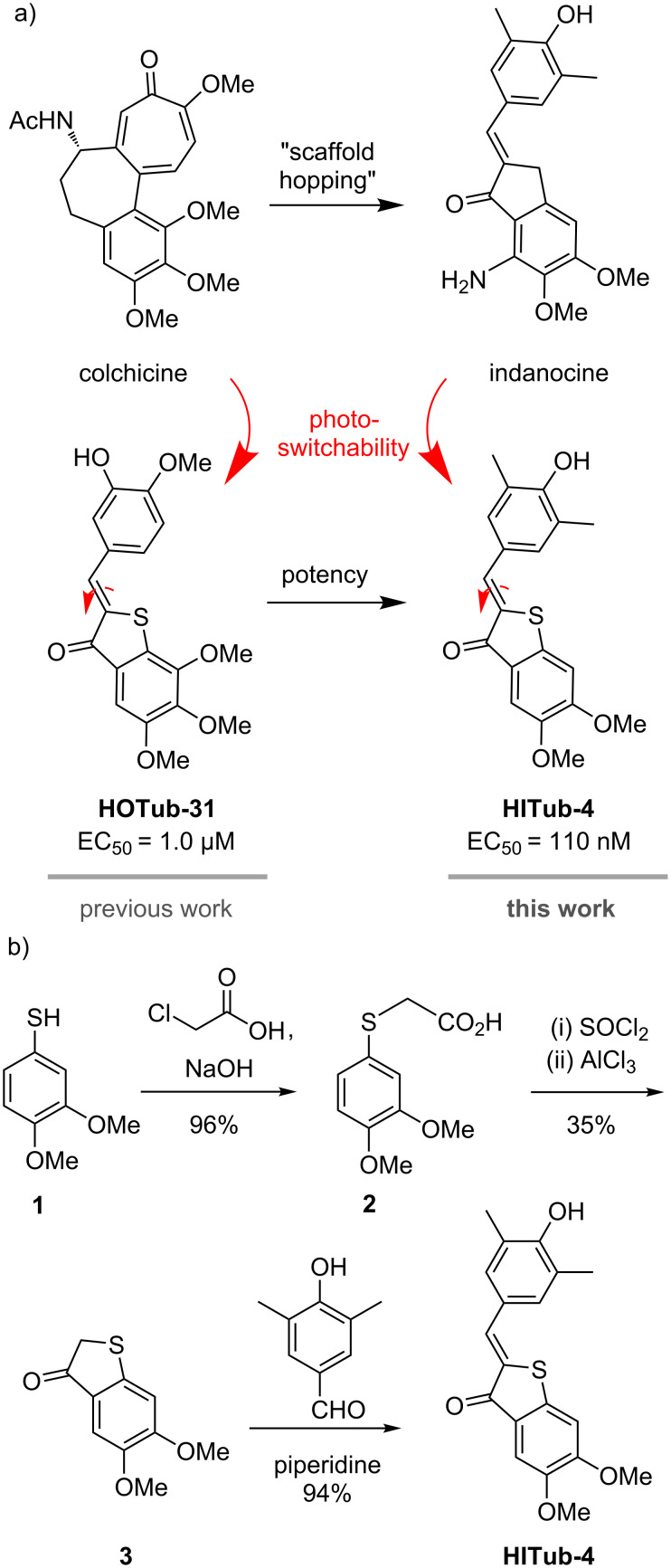
a) The potent tubulin inhibitor colchicine as a lead scaffold led to the development of the **HOTub** generation of HTI-based antimitotics (e.g., **HOTub-31**). Changing the lead scaffold to indanocine led to the development of up to ten times more potent **HITubs** (e.g., **HITub-4**). b) Straightforward, short, and high-yielding synthesis of **HITub-4**.

We and others have reported on photoswitchable azobenzene-based inhibitors of tubulin polymerisation [10–13] that have since been used in studies of neuronal trafficking [14] and embryonic development [15–16], and we have recently reported biologically robust heterostilbenes that deliver green fluorescent protein (GFP)-orthogonal MT photocontrol [17]. However, in both azobenzene and heterostilbene scaffolds, the steric properties of the *E*- and *Z*-isomer are so different that the protein binding site shape determines that the *Z*-isomer (the lit-form) is the more bioactive one, without the possibility of sign inversion by substituent shifts. To overcome this conceptual limitation, we recently reported on the first use of hemithioindigos (HTIs) as photoswitchable pharmacophores for optical control of tubulin dynamics in vitro (cell-free) and MT-dependent processes in cellulo [18]. We showed for the first time that the pseudosymmetry of hemithioindigos can be used to enable a priori design of HTI-based pharmacophores for a single binding site, with higher bioactivity as either the lit-form *E*- or the dark-form *Z*-isomer, just by changing substituent patterns, and developed HTI-based antimitotics with cytotoxic potencies in the low micromolar range (Figure 1a) [18].

In this work, we wished to enhance the bioactivity of the distinctive dark-active HTI-based tubulin-binding antimitotics while retaining the benefits of the HTI scaffold, namely robust, fatigue-resistant, all-visible-light photoswitching.

## Results and Discussion

### Design strategy for HTIs

The HTI-based colchicinoid **HOTubs** (e.g., **HOTub-31**) that we previously explored had the HTI photoswitch embedded inside a methoxylation pattern, such that one isomer obeyed the structure–activity relationship (SAR) of colchicine or its analogue combretastatin A-4 and was bioactive, while the other isomer clashed with their SAR and was less active [18]. That approach of directly embedding a photoswitch motif inside the pharmacophore seemed to be more promising for photopharmacology than the synthetically more straightforward attachment of photoswitches on the pharmacophore periphery. We expected that embedding (which is referred to as azologization in the case of azobenzene-based photopharmaceuticals [19]) should in general lead to more significant alterations of the binding-relevant structure, and increase the differential potency between isomers of the resulting photopharmaceutical, than peripheral attachment (referred to as azo-extension in the case of azobenzenes [18]). We therefore desired to maintain the embedding strategy, yet to improve potency we chose to break with substitution patterns strictly based on colchicine. It is not the case that colchicine (or any other small-molecule inhibitor) represents an ideal structure that colchicine domain inhibitors (CDIs) should reproduce. Thus, our design focus was to introduce reversible photoresponse to a CDI rather than developing compounds with high similarity to colchicine per se, aiming at compounds where one isomer would be almost biologically inactive such that light can be used to effect a photoisomerisation-based switch-on/switch-off of bioactivity. The end-to-end distance of the HTI scaffold is significantly longer than that in either the biaryl colchicine or the stilbene combretastatin, and the torsion angle between the aryl blades of the HTI is nearly planar (up to 4°), while that between the rings of (*Z*)-combretastatin or colchicine is approximately 50–60° [20]. Thus, we assumed that the length and the near-planarity of the HTI could suit it to different substituent patterns to those of colchicine, and attempted to rationally determine these.

Firstly, since the HTI scaffold is longer than a biaryl motif but should occupy a similar volume in the pocket, we assumed that we would have to reduce the substituent bulk present on colchicine/combretastatin A-4. The middle methoxy group of colchicine’s trimethoxy-substituted “south ring” (Figure 1a) makes a beneficial polar contact in the binding pocket via the oxygen atom, but upon demethylation, the potency is much reduced, presumably from insufficient desolvation in the colchicine site (which is known from work on podophyllotoxin derivatives [20]). We therefore chose to keep that methoxy group intact. However, colchicine’s methoxy group on the "north ring" establishes a non-polar spacefilling interaction, which can be replaced equipotently by an ethyl group. Thus, we considered that the HTI scaffold could best be reduced in volume by "shortening" this substituent, maintaining the abovementioned non-polar interaction.

Secondly, since the torsion angle of the HTI is far lower than that of (*Z*)-stilbenes or biaryl compounds, we considered that even with shortening, their SARs might not directly match. Mainly, we assumed that re-orientation of the substituent pattern on one or both rings (e.g., re-orientation of the archetypal 3,4,5-trimethoxyphenyl south ring pattern to a 4,5,6-trimethoxyaryl pattern) might be needed to occupy a similar space.

A wealth of CDIs have been reported, including scaffolds such as aurones that apparently reproduce the substituent pattern SAR of combretastatins [21] while having closely similar scaffold steric properties to HTIs. However, in light of the considerations above, we rather selected indanocine as a starting point for alternative substituent patterning (Figure 1a). Indanocine is a cytotoxic indanone-based CDI (EC_50_ ≈ 10–40 nM) [22] with similar cell culture potency to colchicine (EC_50_ ≈ 3–20 nM) [23] that likewise disrupts MTs, arrests cells in the G2/M phase, and induces apoptosis. Although the size and geometry of thioindoxyl and indanone rings differ because of the S/CH_2_ replacement, we assumed that "mapping" the substitution pattern of indanocine onto a hemithioindigo core should result in a lead structure for tubulin-binding (*Z*)-HTIs, namely the class of **HITubs**.

The *para*-hydroxy substitution of indanocine suggested that HTI might be a more desirable photopharmaceutical scaffold than the widely used azobenzene motif. While *para*-hydroxyazobenzenes feature negligibly short *cis*-to-*trans* thermal relaxation half- lives in aqueous media in the range of μs [24–25], probably making them unsuitable for robust photoswitching applications against intracellular targets, data for *para*-hydroxy HTIs have not been reported so far. We considered that if the *para*-hydroxy-**HITubs** featured photoswitchable bioactivity in cellulo, implying suitable (*E*)-HTI stability under cellular conditions, this would more generally commend them as a scaffold of choice for cellular photopharmaceutical use with strong electron-donating substituents, such as amino or hydroxy groups in *ortho*- or *para*-position, aiming at intracellular targets. This is an important scope of substituents to address, since these small polar groups often establish high-affinity ligand–target interactions, but otherwise represent an obstacle to photoswitchability with azobenzene compounds.

### Preview: design of target **HITubs**

**Figure 2:**
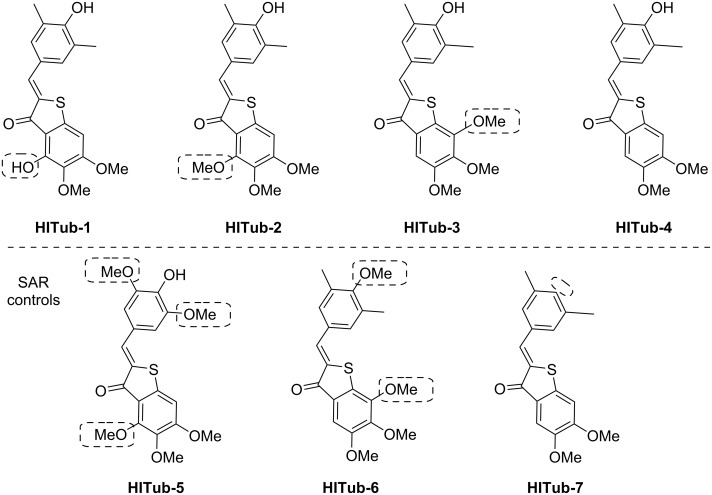
Chemical structures of **HITubs**. Key variations with respect to **HITub-4** are highlighted in dashed boxes.

Our SAR-driven compound development path is described in full later in the section on bioactivity, but in brief, we began the series of indanocine-inspired HTI designs by replacing the south ring amino unit of indanocine (which is attached in *ortho*-position to the key south ring methoxy group) by a hydroxy function, giving **HITub-1** (Figure 2).

When **HITub-1** later proved less bioactive than we had wished, we explored steric and polarity changes to this south ring hydroxy group by methylation (**HITub-2**), methylation and shifting on the ring (**HITub-3**), or even its removal (**HITub-4**). We additionally controlled against our design logic of north ring substituent shortening (**HITub-5**). We also controlled for the SAR observation that CDIs should not tolerate a non-polar central north ring substituent (**HITub-6**), but can support removal of this substituent altogether, with only small potency loss (**HITub-7**) [20]. The progression and results of this SAR study are explained below in the section on bioactivity.

### General synthetic access

Synthetic routes to HTIs are well established [26] and typically involve aldol condensation of benzaldehyde onto thioindoxyls. However, the key step is the formation (and where necessary, isolation) of the thioindoxyl species. In our studies, the electron-rich dimethoxy- and trimethoxy-substituted thioindoxyls were noted to be unstable to air, base, and silica gel during chromatography, so it was sought to minimise their exposure to these conditions during synthesis. In the end, we used two routes to the thioindoxyls: either Friedel–Crafts acylation of α-phenylthioacetic acids (which are easily accessible from thiophenols by alkylation using 2-chloroacetic acid, Figure 1b) or else, lithium diisopropylamide (LDA)-mediated cyclisation of 2-(methylthio)benzamides, which were obtained by directed *ortho*-metalation of the respective benzamides followed by quenching with dimethyl disulfide [27] (Supporting Information File 1, Scheme S1). In general, we found the LDA-mediated cyclisation more convenient, as it generated fewer side products and enabled faster, easier workup and purification. We used these routes to synthesise the **HITubs** typically in good (32% for **HITub-4**, Figure 1b) to excellent (93% for **HITub-7**, Scheme S5) overall yields from commercial building blocks (see Supporting Information File 1).

### Photocharacterisation

Although some *para*-hydroxy-substituted HTIs have been described [28–29], we are unaware of any report of the solvent- and pH-dependency of their photochromism and thermal relaxation. Dube and co-workers have reported that in general, increasing the electron-donating strength of groups in the hemistilbene *para*-position of HTIs correlates to (a) a bathochromic shift of the S_0_ → S_1_ absorption band (up to λ_max_ ≈ 500 nm with julolidine substitution) and (b) decreased thermal stability of the metastable *E*-isomer, i.e., faster thermal relaxation [30]. However, they also reported that introducing electron-donating groups (methoxy, dimethylamino) in *para*-position to the thioindoxyl sulfur atom restored *E*-stability while maintaining red-shifted absorption maxima. This *para*-position was occupied by the key methoxy group in all our **HITub** designs. With scant information available, we could not predict the thermal stability of (*E*)-**HITubs** in cellular conditions, so we turned to experimental measurement.

**Figure 3:**
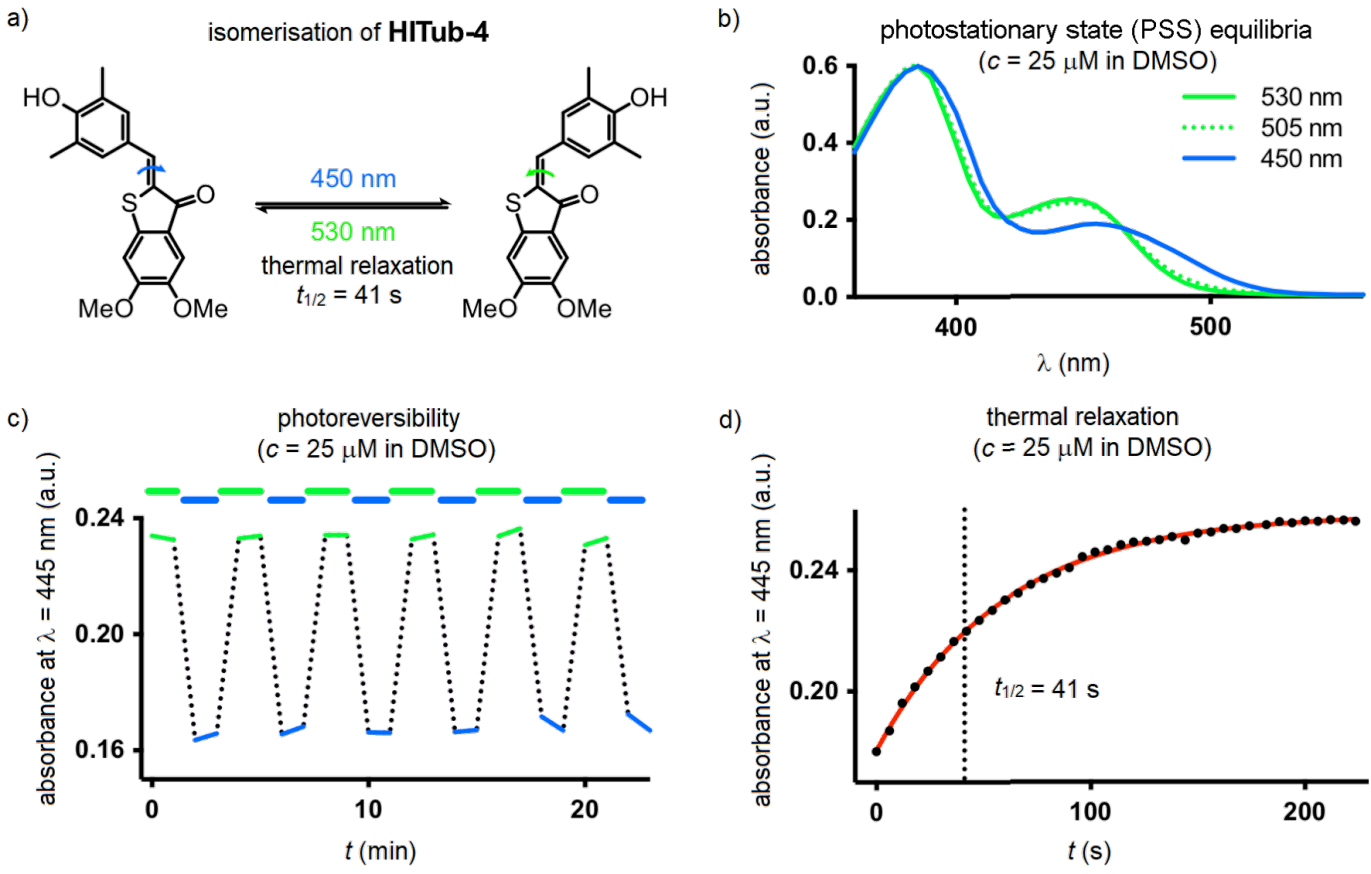
Photocharacterisation of **HITub-4**. a) Photochemical and thermal isomerisation. b) UV–vis spectra after saturating illumination at λ = 450, 505, and 530 nm, respectively. c) Photoreversible switching upon alternating illumination (λ = 450 and 530 nm). d) Thermal relaxation at room temperature after reaching the photostationary state at λ = 450 nm.

Since we found no substantial differences between the photochemical properties of the *para*-hydroxylated compounds (Figure 3 and Supporting Information File 1, Figure S1), we here describe the photocharacterisation of **HITub-4** as a representative example of the photoswitchable bioactive compounds (for more detailed analysis see Supporting Information File 1). In polar aprotic solvents, the **HITub-4** *Z*-isomer (λ_max_ ≈ 380, 460 nm) showed robust, reliable, and fully reversible photoswitching (λ = 450 nm for *Z* → *E* and 530 nm for *E* → *Z* switching), with the high fatigue resistance characteristic of HTIs. The *E*-isomer's thermal half-life in EtOAc or DMSO was ca. 40 s (Figure 3 and Supporting Information File 1, Figure S3). Its absorption spectra and photoswitchability were unaltered by the addition of acid, however, addition of base led to a remarkable bathochromic and hyperchromic shifts of the absorption band at ca. 550 nm, and no observed photoswitchability (Supporting Information File 1, Figure S2). We assumed that this spectrum resulted from a quinoidal structure, formed after deprotonation of the hydroxy group, and that the lack of observable photoswitchability arose due to fast free rotation around the C–C single bond connecting the thioindigo and hemistilbene motifs. Interestingly, in neutral or acidic aqueous media where the quinoidal structure is not present (λ_max_ ca. 370, 480 nm), photoswitching could not be observed either, which we presumed to be due to fast thermal relaxation. However, noting that typical CDIs are substantially biolocalised into lipid environments within cells [31], we decided to explore photoswitching-based cellular assays with these compounds nonetheless (further discussion in Supporting Information File 1).

We found that the photochemical properties of the non-*para*-hydroxylated control **HITub-6** were similar to those of previously reported non-*para*-hydroxylated **HOTubs** [18], with satisfactory photoswitching in both DMSO and phosphate-buffered saline (PBS)/DMSO mixtures (Figure S1f,g in Supporting Information File 1).

### Bioactivity: SAR study of **HITubs** in cellulo

**Figure 4:**
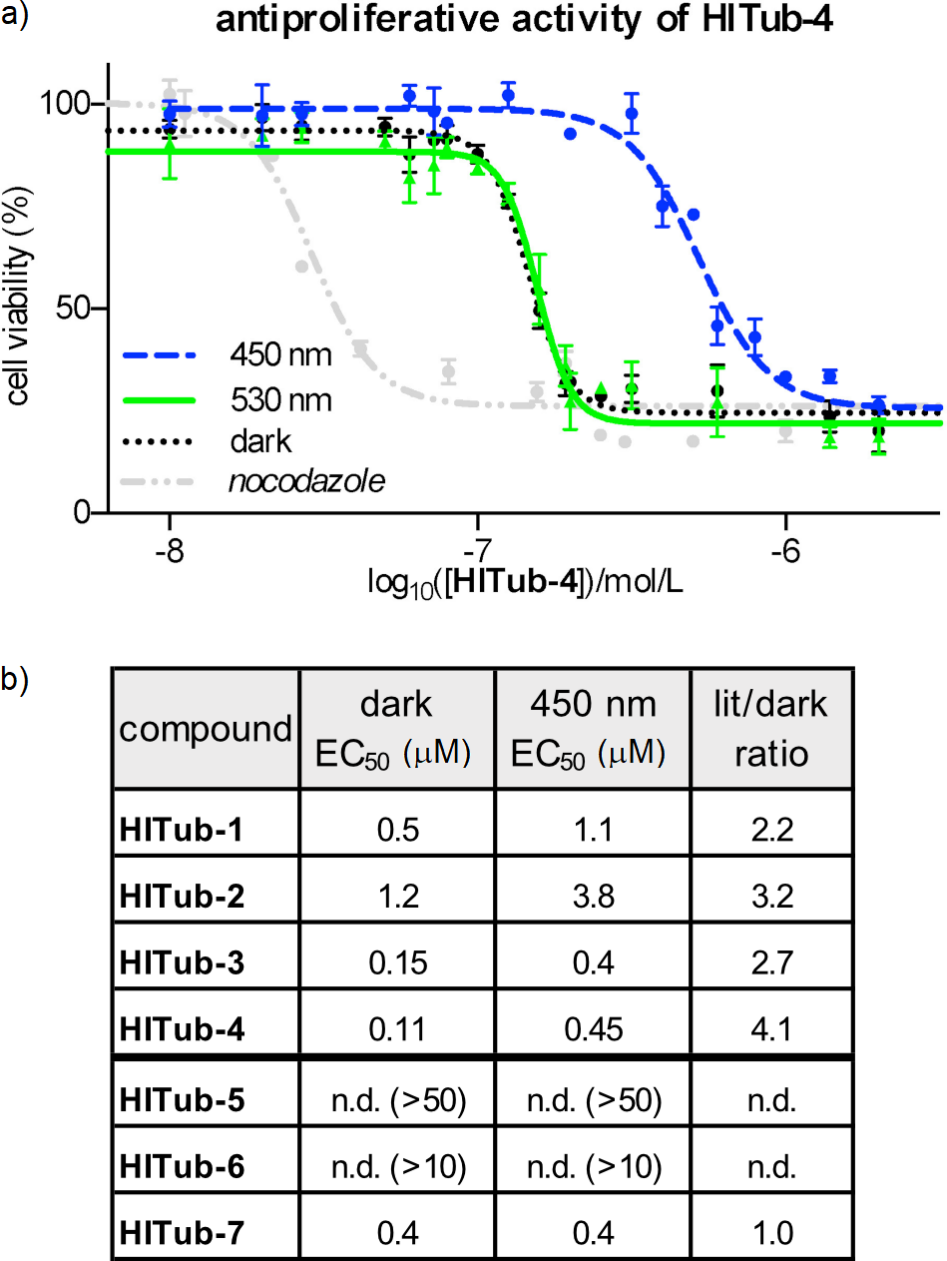
a) Resazurin reduction assay for **HITub-4** and nocodazole in HeLa cells (*n* = 3), demonstrating the difference in antiproliferative potencies at λ = 450 and 530 nm/dark conditions (75 ms pulsing every 15 s). b) EC_50_ values of **HITubs** from cellular antiproliferation assays under dark conditions and at λ = 450 nm. The ratio of lit/dark EC_50_ values shows the fold change in photoswitchable bioactivity.

To begin evaluating the isomer-dependent bioactivity of **HITub** photopharmaceuticals in cellulo, we first performed resazurin (resorufin *N*-oxide) antiproliferation assays under different lighting conditions (Figure 4). Inhibitors of tubulin polymerisation act as antimitotic cytotoxins in cell culture by preventing formation of a functional mitotic spindle, resulting in mitotic arrest and eventually cell death. The reduction of resazurin by viable cells serves as a fluorogenic proxy readout for antimitotic potency in cellulo, since the degree of resazurin turnover scales to the number of cells still viable after compound treatment, although the mechanism behind antiproliferative activity must later be determined using more specific assays. We used the HeLa human cervical cancer cell line to assess the biological activity of **HITubs** in all cellular experiments shown in this work. Since tubulin is a highly conserved protein target critical for survival in all cell types, we expected that, as for other colchicine domain tubulin inhibitors, trends in potency and in photoswitchability of potency determined in this representative mammalian cell line can be translated to other cell types, although their specific response (e.g., EC_50_ values) would need individual determination. Nocodazole was used as a benchmarking reference and mechanistic positive control in all in cellulo assays since it is a potent inhibitor of the colchicine binding site (EC_50_ ≈ 40 nM) with both appropriate solubility and straightforward handling.

Self-made low-intensity LED arrays with relatively narrow bandwidth were used for illumination of cells during assays, with a pulsing regime of 75 ms every 15 s to maintain photostationary state equilibria in cellulo [10]. We cross-checked different illumination wavelengths in cellular toxicity assays; in accordance with the DMSO photoswitching studies, we observed that 530 nm (ca. 97% *Z*-configuration, but additionally controls for non-specific phototoxicity) delivered results equivalent to dark conditions (exclusively *Z*-configuration), to which 450 nm (lit conditions, ca. 70% *E*-configuration) gave the greatest difference in antiproliferative potencies.

We began our studies with **HITub-1**. This is a HTI analogue of indanocine in which the indanocine amino function (in *ortho*-position to the key south ring methoxy group) has been replaced by a synthetically more accessible hydroxy group (delivered via demethylation of a trimethoxy precursor through BBr_3_). The hydroxy and amino groups have similar size and polarity, and can both act as H-bond donors or acceptors. Therefore, we expected **HITub-1** to allow reliable evaluation of the indanocine substituent pattern. (*Z*)-**HITub-1** was already strongly bioactive (EC_50_ ≈ 500 nM, Figure 4b), although one order of magnitude less so than indanocine (EC_50_ ≈ 10–40 nM, depending on the cell line). Pleasingly, although we had not observed its photoswitching in pure aqueous media, in the heterogeneous cellular environment, we found that its overall toxicity under lit conditions was reliably and reproducibly halved.

We then explored changes to the substituent pattern to determine whether we could improve both *Z*-isomer potency in an absolute sense and the overall photoswitchability of potency comparing *E*- and *Z*-isomers. We began by methylating the south ring hydroxy group (**HITub-2**) to see how changes in size and polarity affect the bioactivity; surprisingly, the potency loss was not dramatic (indicating that this position is not a key determinant of bioactivity), but the photoswitchability increased substantially (3-fold with regard to the lit/dark ratio). We took this as an encouraging indicator of the overall polarity required for binding, and now examined re-orienting the south ring substituents by shifting the trimethoxy pattern on the ring (**HITub-3**), which improved the potency dramatically (EC_50_ ≈ 150 nM for the *Z*-isomer) while retaining the 3-fold photoswitchability of bioactivity.

Since comparison of **HITub-2** and **HITub-3** showed that the potency can be retained without substituents in *ortho*-position to either the carbonyl group or the sulfur atom, for maximal simplicity, we tested whether both substituents could be deleted simultaneously (**HITub-4**). This proved to be the strongest-performing compound of our studies, with the *Z*-isomer possessing an EC_50_ value of ca. 110 nM and a 4-fold difference of bioactivity between lit and dark conditions (λ = 450 nm). This difference was surprisingly high, given that even in aprotic media (e.g., lipid environment reservoirs within cells), there should be ca. 30% residual *Z*-isomer at photoequilibrium [18], and we had expected that any (*E*)-**HITub** entering the cytosol (aqueous environment) would quickly relax to its more bioactive *Z*-isomer before encountering its cytosolic protein target. We theorised that fast cytosolic relaxation of the *para*-hydroxy **HITubs** to their bioactive *Z*-isomer may actually be a decisive factor in preventing the simple equilibration of the extracellular **HITub** concentration (exclusively *Z*-configuration due to fast relaxation, irrespective of illumination conditions) with the cytosolic (*Z*)-**HITub** concentration available to bind to tubulin (and which all experiments show is reduced under λ = 450 nm illumination). Examining this in detail is beyond the scope of this study, however, see Supporting Information File 1 for a discussion on isomer-dependent subcellular biolocalisation effects.

We first controlled against our design logic of north ring substituent shortening by changing the north ring apolar-contact methyl groups to methoxy groups (**HITub-5**), and were satisfied when this abolished bioactivity. We also controlled for the result, known from extensive SAR work at the colchicine site [18,32], that CDIs should not tolerate a non-polar central north ring substituent (**HITub-6**) but can support removal of this substituent altogether, with only small potency loss (**HITub-7**). We considered that if the screened compounds obeyed this principle, it would reinforce our mechanistic understanding of them as CDIs. Indeed, **HITub-6** proved inactive until it reached its solubility limit, but (*Z*)-**HITub-7** was relatively potent and featured an only 4-fold reduction of bioactivity as compared to its hydroxylated parent (*Z*)-**HITub-4**. Interestingly, however, **HITub-7** displayed no difference between dark (all-*Z*) and lit (mostly *E*) conditions, and we were unable to rationalise this with reference to either polarity or structure, in light of our prior work on apolar **HOTubs** [18] (see also Supporting Information File 1). We were, however, overall satisfied by these findings (Supporting Information File 1, Figure S4), especially by the potency and photoswitchability of **HITub-4**.

These results indicated that indanocine-inspired HTI-based reagents are a potent, cellularly bioactive class of photoswitchably antiproliferative agents, with the most potent light-controlled antimitotic bioactivity reported for photoswitches designed for tubulin: 10-fold enhancement compared to the predecessor HTI generation **HOTubs** [18] and styrylbenzothiazole-based **SBTubs** [17], and 5-fold superior to azocombretastatins [10–12]. In view of the generally limited solubility of photopharmaceuticals (associated with their extended flat aromatic structures), this increase in potency renders the **HITub** compound class a promising addition to the toolbox of photoswitchable antimitotics, which might prove valuable for future in vivo studies.

We also noted that the *para*-hydroxy **HITubs** featured ca. 30% residual (*Z*)-**HITub** at PSS λ = 450 nm in cell-free measurements, and that for **HITub-2**–**4**, the PSS isomer mixture's cellular cytotoxicity at that wavelength was on average 3.3-fold lower than the cytotoxicity of the corresponding (*Z*)-**HITub** (Figure 4). This can be interpreted as indicative that the *E*-isomers are essentially biologically inactive, similar to what has been observed for heterostilbene **SBTubs** [17] and azobenzene photostatins (**PSTs**) [10]. If substantiated, HTI-like analogues for which photostationary state (PSS) with enhanced proportions of *E*-isomer can be photogenerated would represent an exciting advance: they could, in contrast to the other photoswitch types, allow all-visible, photoreversible, high-potency switching while reproducing similarly beneficial photoswitchability of bioactivity.

### Mechanistic assessment of **HITub** action

**Figure 5:**
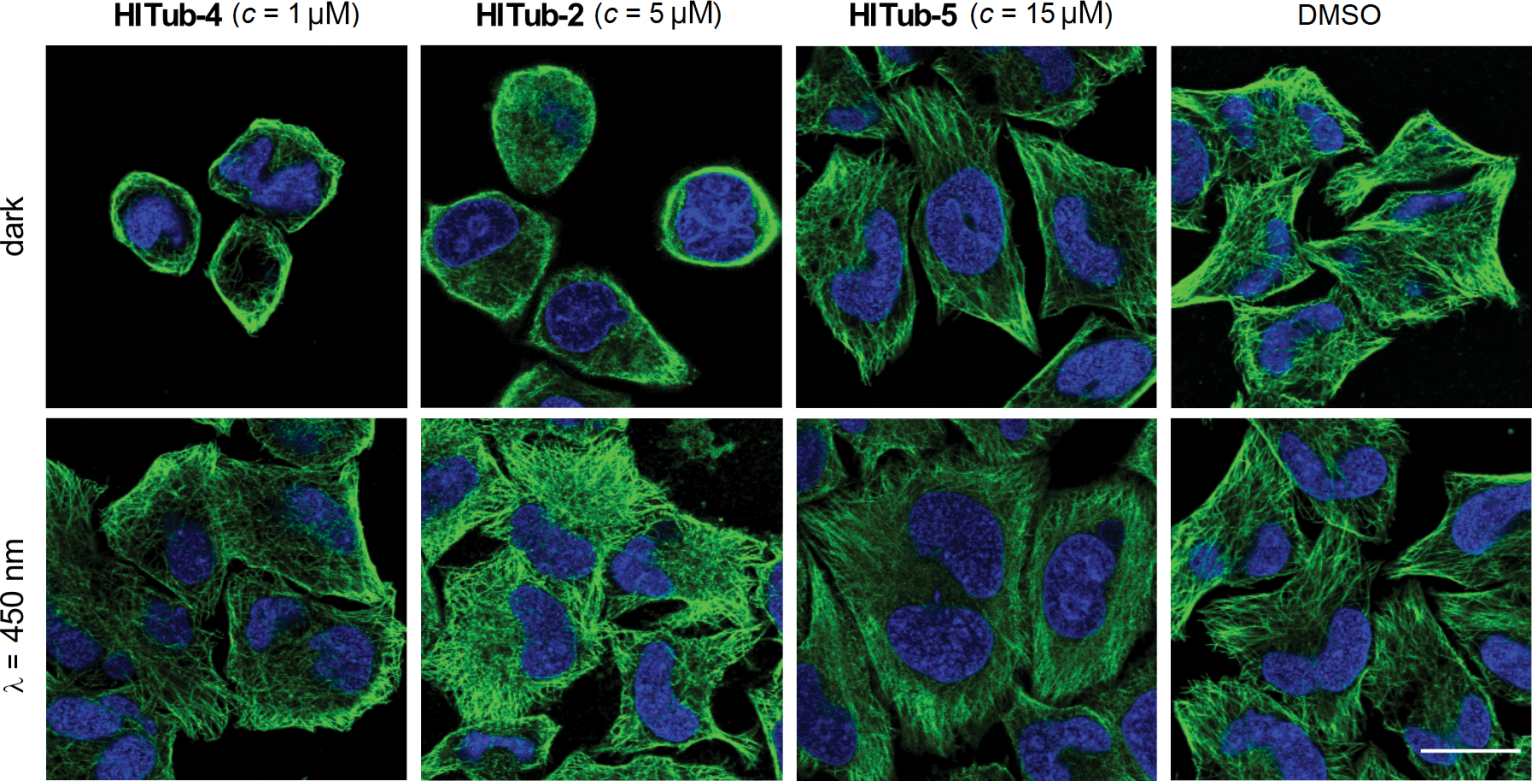
Confocal microscopy images of immunofluorescently labelled MT networks after treatment with **HITubs** for 24 h under lit (λ = 450 nm) and dark conditions. **HITub-4** and **HITub-2** caused light-dependent MT disruption at low concentrations (*c* = 1 and 5 μM, respectively) matching their cytotoxicities (EC_50_ = 1 and 5 μM, respectively). High concentrations of the structurally related negative control **HITub-5**, like the DMSO co-solvent control, produced no effects under either illumination condition. MTs (anti-α-tubulin) are visualized in green while the nuclear stain 4′,6-diamidino-2-phenylindole (DAPI) is visualized in blue. Scale bar length = 20 μm.

We now determined to confirm the mechanism of action of the **HITub** compounds. To evaluate the biological mechanism of action behind the **HITubs**’ photoswitchable antimitotic activity, we first checked their inhibition of polymerisation of purified tubulin in a cell-free assay. The results showed almost identical inhibition potency for **HITub-4** at *c* = 10 μM as for the archetypal CDI colchicine at *c* = 20 μM (Figure S5, Supporting Information File 1), which we took to indicate that (*Z*)-**HITub-4** exerted its bioactivity by specifically binding to tubulin directly in the cell-free system. This suggests that the same specific direct action can be reproduced in cellulo, and that effects on auxiliary cellular systems dependent upon the MT cytoskeleton can likely be downstream effects of MT depolymerisation. We next investigated the **HITubs**’ isomer-dependent effects on the MT network inside cells, focusing on the active analogues **HITub-4** and **HITub-2** in comparison with inactive **HITub-5** as a control (Figure 5). By reducing tubulin polymerisation dynamics, CDI treatment should first disorganise and then depolymerise the cellular MT network. We performed immunofluorescence staining of the MT network within cells treated with **HITubs** and documented the resulting disruption of the physiological MT network integrity, and consequently also changes in cell morphology by confocal microscopy. Cells exposed to **HITub-4** (1 μM) in the dark or under λ = 530 nm illumination (to maintain exclusively *Z*-configuration) show near-complete disruption of MT structures after 24 h, while treatment with λ = 450 nm illumination caused no significant disruption of the MT network compared to an untreated dark control (Figure 5). Less potent **HITub-2** also showed similar light dependency of its biological effects at higher concentrations (Supporting Information File 1, Figure S6). Pleasingly, SAR control **HITub-5** showed no impact on MT integrity at the highest tested concentration under lit or dark conditions, which we took as a promising indication for the absence of phototoxicity or of other effects non-specific to tubulin disruption.

**Figure 6:**
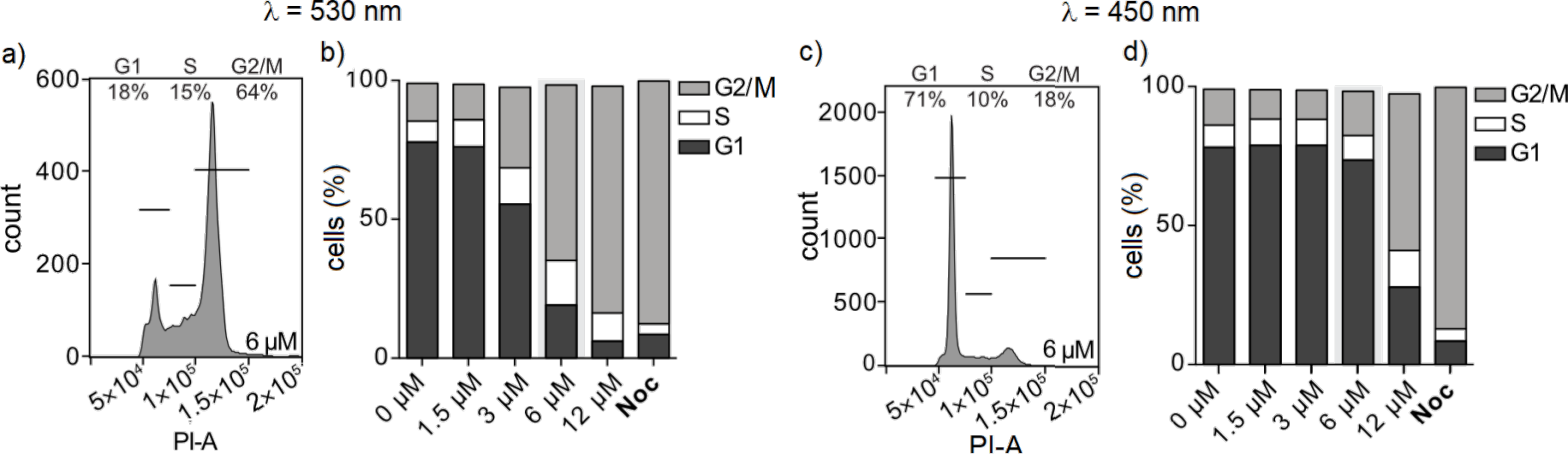
Cell cycle analysis of **HITub-4**-treated cells. a) and b) (*Z*)-**HITub-4** caused significant G2/M arrest already at a concentration of 6 μM (λ = 530 nm). c) and d) Under irradiation at λ = 450 nm, where a majority of (*E*)-**HITub-4** was present, no mitotic arrest could be observed at comparable concentrations. a) and c) Representative histograms showing cell cycle arrest, with population binning as indicated. In b) and d), nocodazole (Noc) was used as a positive control (*c* = 1 μM).

Lastly, to substantiate the causative link between the observations on MT disruption and cellular toxicity, we examined the impacts of **HITub-4** on the cell cycle. Tubulin-binding agents whose major cellular mechanism of toxic action is the disruption of MT dynamics or structure should cause cell cycle arrest in the G2/M phase by preventing the completion of mitosis [1]. We examined cell cycle repartition by quantification of cellular DNA content via propidium iodide (PI) incorporation, which was analysed by flow cytometry (Supporting Information File 1, Figure S7). HeLa cells were treated for 24 h with **HITub-4** under λ = 530 and 450 nm irradiation, respectively, with the synthetic tubulin-binding agent nocodazole (Noc) used as a reference. As expected, **HITub-4** showed highly light-dependent bioactivity with near-complete G2/M phase arrest at a concentration of 6 μM and λ = 530 nm irradiation (Figure 6a and Figure 6b), but nearly no cell cycle interference at the same concentration and λ = 450 nm irradiation (Figure 6c and Figure 6d).

## Conclusion

Taken together, these results indicate that the **HITubs** had achieved their design aims, being a rationally-designed, potency-enhanced set of HTI-based tubulin-inhibiting photopharmaceuticals with photoswitchable bioactivity across cell biology assays, allowing reliable photocontrol over tubulin polymerisation, MT network structure, cell cycle, and cell survival. They feature mid-nanomolar potency in cellulo, the highest yet reported for photopharmaceutical tubulin inhibitors, as well as satisfactory photoswitchability of potency. We expect that due to the **HITubs**’ potency of tubulin inhibition, they will prove a powerful reagent system for biological studies on MT, especially where dark-isomer activity (compared to the currently known, lit-active azobenzenes or styrylbenzothiazoles) is desirable, in particular for cell-free mechanistic studies [33]. More broadly, this work also shows that the HTI scaffold robustly enables the photoswitchable use of resonance-capable substituents that can establish high-affinity interactions (such as *para*-hydroxy groups), which are otherwise problematic for current photopharmaceutical scaffolds to tolerate without loss of photoswitchability. In the broader sense, this is of interest for cell biology and further highlights the potential of HTIs as a pharmacophore scaffold for expanding the scope of cellular photopharmacology.

## Supporting Information

File 1 Full experimental protocols for chemical syntheses, photocharacterisation, biochemistry, and cell biology, including NMR spectra.
