# Supplementary material for: Potent hemithioindigo-based antimitotics photocontrol the microtubule cytoskeleton in cellulo
Source: Beilstein J Org Chem. 2020 Jan 27;16:125–34. doi: 10.3762/bjoc.16.14 (PMC7006478; doi:10.3762/bjoc.16.14)

BEILSTEIN JOURNAL OF ORGANIC CHEMISTRY

Supporting Information

for

Potent hemithioindigo-based antimitotics photocontrol the microtubule cytoskeleton in cellulo

Alexander Sailer, Franziska Ermer, Yvonne Kraus, Rebekkah Bingham, Ferdinand H. Lutter, Julia Ahlfeld and Oliver Thorn-Seshold

Beilstein J. Org. Chem. **2020,** *16,* 125–134. doi:10.3762/bjoc.16.14

Full experimental protocols for chemical syntheses, photocharacterisation, biochemistry, and cell biology, including NMR spectra

License and Terms: This is a supporting information file under the terms of the Creative Commons Attribution License (https://creativecommons.org/licenses/by/4.0). Please note that the reuse, redistribution and reproduction in particular requires that the authors and source are credited.
The license is subject to the *Beilstein Journal of Organic Chemistry* terms and conditions: (https://www.beilstein-journals.org/bjoc)

Authorship Statement: Following the ICMJE guidelines, the authors declare their roles in the present work as follows: A.S. performed chemical synthesis, photocharacterisation and coordinated data assembly, F.E. performed in vitro studies, Y.K. performed in cellulo studies, R.B. performed in vitro tubulin polymerisation assays, F.H.L. performed chemical synthesis, J.A. performed in cellulo studies and coordinated data interpretation and assembly. O.T.-S. designed the concept and experiments, supervised all experiments, coordinated all data and wrote the manuscript with input from all authors.

Table of Contents

Part A: Chemical syntheses S3
Conventions S3
Standard procedures S4
Synthesis of HITub-3 .... S6
Synthesis of HITub-4 .... S8
Synthesis of HITub-2 and HITub-5 .... S10
Synthesis of HITub-1 .... S11
Synthesis of HITub-7 .... S13
Synthesis of HITub-6 .... S14
Part B: Photocharacterisation in vitro S17
UV–vis spectrophotometry of bulk samples S17
PSS spectra of the HITubs S17
Solvent- and pH-dependency of photoisomerisability and relaxation rate S18
Part C: Biochemistry and cellular biology S21
Resazurin antiproliferation assays S22
In vitro tubulin polymerisation assay S22
Immunofluorescence imaging of microtubule network structure S23
Cell cycle analysis S24
Supplemental references S25
Part D: NMR spectra S26

Part A: Chemical syntheses

Conventions

Hemithioindigo geometry and nomenclature: Hemithioindigos (HTIs) are drawn by default in their *Z*-isomeric form. However, this should be understood to imply either or both of the *E* & *Z* forms constituting a given sample depending on light exposure, therefore by default they are also named without *E*/*Z*-designations.

Abbreviations: The following abbreviations are used: *sec*Bu – *secondary* butyl, Bu – butyl, calcd. – calculated, cHx – cyclohexane, DCM – dichloromethane, DIPA – *N*-(propan-2-yl)propan-2-amine, DMSO – dimethyl sulfoxide, EA / EtOAc – ethyl acetate, EtOH – ethanol, Hx – distilled isohexanes, HRMS – high-resolution mass spectrometry, LDA – lithium diisopropylamide, MeCN – acetonitrile, *n*-Bu – *normal* butyl, MS – molecular sieves, NIS – N-iodosuccinimide, TFA – trifluoroacetic acid, THF – tetrahydrofuran, TMEDA – tetramethylethylendiamine,.

Automated flash chromatography: Automated flash chromatography was performed on a Biotage Isolera One instrument using 254 nm as detection wavelength and eluting with mixtures of ethyl acetate and isohexanes.

Reagents and conditions: Unless stated otherwise: (1) all reactions and characterisations were performed with unpurified, undried, non-degassed solvents and reagents, used as obtained, under closed air atmosphere without special precautions unless stated otherwise. The use of dry solvents in this context is to be understood as using anhydrous solvents bought from Acros Organics which were stored and handled under an atmosphere of nitrogen; (2) "hexane" used for chromatography was distilled from commercial crude isohexane fraction on rotavap; (3) when not specified, "column" and "chromatography" refer to flash column chromatography performed on Merck silica gel Si-60 (40–63 µm); (4) procedures and yields are unoptimised; (5) yields refer to isolated chromatographically and spectroscopically pure materials (6) all eluent and solvent mixtures are given as volume ratios unless otherwise specified, thus "1:1 EA:Hx" indicates a 1:1 mixture (by volume) of ethyl acetate and hexanes.

Thin-layer chromatography (TLC) was run on 0.25 mm Merck silica gel plates (60, F-254). UV light (254 nm) was used as a visualising agent. R_f values were usually determined in ethyl acetate : hexane (EA:Hx) eluents. TLC characterisations are thus abbreviated as per (R_f = 0.09 on EA:Hx, 6:1).

NMR: Standard NMR characterisation was by 1D ^{1}H- and ^{13}C-NMR spectra, with COSY, HSQC, HMBC or heteronuclear NMR performed as needed. Known compounds were checked against literature data and their spectral analysis is not detailed unless necessary. The default spectrometer used was a Bruker Ascend 400 (400 MHz & 100 MHz for ^{1}H and ^{13}C

respectively); NMR solvents are given individually for each compound. For determination of the composition of the photostationary states (450 and 505 nm), a Bruker Avance III HD (500 MHz) equipped with CryoProbe™ Prodigy broadband probe was used.

Chemical shifts (δ) are reported in ppm calibrated to residual non-perdeuterated solvent as an internal reference[1]. The following peak descriptions are used: singlet (s), doublet (d), triplet (t), quartet (q), multiplet (m), broad (br.).

Mass Spectra: HRMS was carried out by the Zentrale Analytik of the LMU, Munich using ESI or EI ionisation as specified.

Standard procedures

Where standard procedures were used in synthesis, unless stated otherwise, the amounts of reactants/reagents employed were implicitly adjusted to maintain the same molar ratios as in the given procedure, and no other alterations from the standard procedure (e.g., reaction time, extraction solvent, temperature) were made, unless stated otherwise.

Standard procedure A: amidation of benzoic acids

The carboxylic acid was dissolved in an excess of thionyl chloride under an atmosphere of nitrogen and heated to reflux for 3 h. The volatiles were removed under reduced pressure using an external cooling trap, the oily residue was dried in high vacuum and dissolved in dry THF. The solution was cooled to 0 °C and diethylamine (3.00 equiv) was added dropwise via syringe under a stream of nitrogen (a second needle was used as overpressure valve to remove HCl). After complete addition, the cooling bath was removed and the reaction mixture was stirred overnight at room temperature. Water was added carefully, the phases were separated and the aqueous phase was extracted with EtOAc. The combined organic extracts were washed with HCl (2 M, 2 times) and NaOH (2 M, 2 times) to remove residual amine and benzoic acid, respectively. After washing with brine (2 times), the combined organic extracts were dried over Na_2SO_4, evaporated and purified by flash chromatography to yield the benzamide.

Standard procedure B: directed *ortho* metalation and thiomethylation

TMEDA was distilled over KOH pellets under an atmosphere of nitrogen (bp. 123 °C; ambient pressure) and stored under nitrogen. To a solution of TMEDA (1.20 or 2.50 equiv) in dry THF was added *sec*BuLi (1.4 M in cyclohexane, 1.2 or 2.5 equiv) at −78 °C and the bright yellow reaction mixture was stirred for 15 min. prior to dropwise addition of a solution of the benzamide (1.00 equiv) in dry THF. The colourless solution was kept at −78 °C for further 15 min. and a solution of dimethyl disulfide (2.00 equiv) in dry THF was added dropwise. After 15 min. the reaction mixture was allowed to warm to room temperature and stirred for the time indicated. The reaction was quenched by the careful addition of a saturated aqueous solution

of NH_4Cl, the phases were separated and the aqueous phase was extracted with EtOAc (3 times). The combined organic extracts were washed with brine (3 times), dried over Na_2SO_4 and evaporated. The crude was purified as stated to yield the *ortho*-thiomethyl benzamide.

Standard procedure C: base-mediated cyclisations of *ortho*-thiomethyl benzamides

LDA was prepared fresh by adding n-BuLi (2.50 M in cyclohexane) to a solution of DIPA in dry THF. To a solution of LDA (1.50–4.30 equiv) in dry THF was added the *ortho*-thiomethyl benzamide (1.00 equiv) at −78 °C and the reaction mixture was stirred at this temperature for 1–1.25 h, then allowed to warm to room temperature and stirred for another hour. The reaction was quenched by the careful addition of a saturated aqueous solution of NH_4Cl, the phases were separated and the aqueous phase was extracted with DCM. The combined organic extracts were dried over Na_2SO_4 and evaporated. Purification was accomplished by (automated) flash chromatography to yield the thioindoxyl.

Standard procedure D: formation of HTIs via piperidine catalysed aldol condensations

An oven dried Schlenk flask was charged with the thioindoxyl (1.00 equiv), the aldehyde (equivalents are given individually) and molecular sieves (4 Å) under an atmosphere of nitrogen. Dry benzene or toluene and piperidine (few drops) were added and the solution was heated to 80 °C for the time indicated individually. After cooling to room temperature, a saturated aqueous solution of NH_4Cl was added, the phases were separated and the aqueous layer was extracted with DCM. The combined organic extracts were dried over Na_2SO_4 and evaporated. Purification was accomplished as stated to yield the hemithioindigo.

Standard procedure E: formation of HTIs via dehydrative ring closure

The (phenylthio)acetic acid (1.00 equiv) was suspended in an excess of Eaton's reagent (P_2O_5 in methanesulfonic acid) and stirred for 2 h at 50 °C. The reaction was quenched by addition of water and the mixture was extracted with DCM. The combined organic layers were washed with a saturated aqueous solution of $NaHCO_3$ (3 times), brine, dried over Na_2SO_4 and the volume was reduced to 5–6 mL. To this solution of thioindoxyl was added basic Al_2O_3 and the corresponding aldehyde (typically 0.25–0.45 equiv; as stated) and the mixture was stirred at ambient temperature. After completion of the reaction (detected by TLC), the mixture was filtered through a silica gel pad on 1:1 EA:Hx and the solvent was removed under reduced pressure. Purification was accomplished by flash chromatography, preparative thin-layer chromatography or precipitation as stated to yield the hemithioindigo.

Synthesis of HITub-3

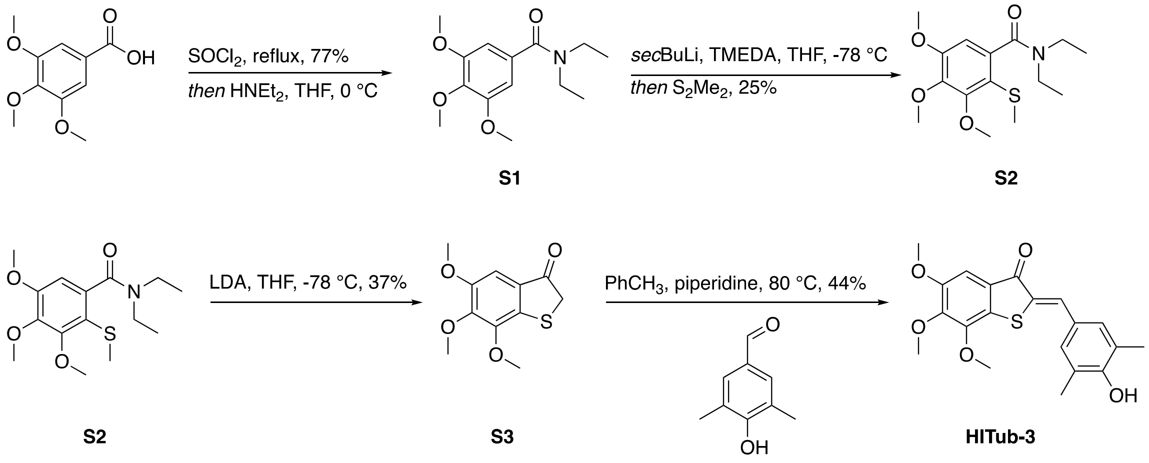

Scheme S1: Synthesis of **HITub-3**.

***N*,*N*-Diethyl-3,4,5-trimethoxybenzamide (S1)**

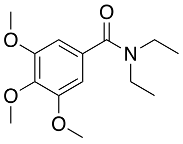

By standard procedure A, reacting commercially available 3,4,5-trimethoxybenzoic acid (1.00 g, 4.71 mmol, 1.00 equiv) with excess thionyl chloride (5 mL) followed by treatment of the acid chloride with diethylamine (1.03 g, 14.1 mmol, 1.46 mL, 3.00 equiv) in dry THF (20 mL). Purification by means of flash chromatography (EA:Hx, 1:1 → 3:1) afforded amide **S1** (969 mg, 3.62 mmol, 77%) as colorless oil that solidified upon standing.

The spectral data match the literature[2].

^{1}H-NMR ($CDCl_3$, 400 MHz): δ (ppm) = 6.56 (pseudo d, 2H), 3.84 (pseudo d, 6H), 3.82 (pseudo d, 3H), 3.50 (br. pseudo s, 2H), 3.27 (br. pseudo s, 2H), 1.20 – 1.13 (br. m, 6H, H); **^{13}C-NMR** ($CDCl_3$, 100 MHz): δ (ppm) = 171.1, 153.4, 138.7, 132.8, 130.0, 103.6, 61.0, 56.3, 43.4, 39.4, 14.5, 13.0; **R_f** = 0.20 on EA:Hx, 1:1; ESI$^+$ **HRMS** for $C_{14}H_{22}NO_4^+$: calcd. m/z 268.15433, found m/z 268.15450; for $C_{14}H_{21}NO_4Na^+$: calcd. m/z 290.13628, found m/z 290.13640.

***N*,*N*-diethyl-3,4,5-trimethoxy-2-(methylthio)benzamide (S2)**

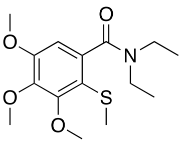

By standard procedure B, reacting amide **S1** (640 mg, 2.39 mmol, 1.00 equiv) with *sec*BuLi (3.21 g, 5.99 mmol, 4.28 mL, 2.50 equiv) and TMEDA (696 mg, 5.99 mmol, 0.90 mmol, 2.50 equiv) followed by quenching with dimethyl disulfide (451 mg, 4.79 mmol, 0.43 mL,

2.00 equiv) in dry THF (20 mL) overnight. Purification was accomplished by flash chromatography (EA:Hx, 1:1) and automated flash chromatography (EA:Hx, 5 → 60%) affording **S2** as a colorless oil (184 mg, 0.59 mmol, 25%).

The analytical data is in good agreement with our previously published data[3].

^{1}H-NMR ($CDCl_3$, 400 MHz): δ (ppm) = 6.39 (s, 1H), 3.81 (s, 3H), 3.72 (s, 3H), 3.70 (s, 3H), 3.66 – 3.59 (m, 1H), 3.25 – 3.17 (m, 1H), 3.01 – 2.96 (q, *J* = 7.1 Hz, 2H), 2.21 (s, 3H), 1.12 (t, *J* = 7.1 Hz, 3H), 0.92 (t, *J* = 7.1 Hz, 3H); **^{13}C-NMR** ($CDCl_3$, 100 MHz): δ (ppm) = 168.8, 155.1, 154.1, 142.4, 138.0, 117.7, 104.8, 61.0, 60.7, 55.9, 42.6, 38.5, 19.1, 13.8, 12.3; **R_f** = 0.36 on EA:Hx, 1:1; ESI$^+$ **HRMS** for $C_{15}H_{23}NO_4SNa^+$: calcd. m/z 336.12400, found m/z 336.12402.

5,6,7-Trimethoxybenzo[*b*]thiophen-3(2*H*)-one (S3)

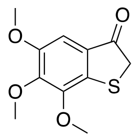

By standard procedure C, **S2** (25 mg, 79.8 µmol, 1.00 equiv) was reacted with LDA (prepared from *n*-BuLi (36 mg, 120 µmol, 0.05 mL, 1.50 equiv) and DIPA (20 mg, 200 µmol, 0.03 mL, 2.50 equiv)). After stirring for 1 h at −78 °C and 1 h at ambient temperature, the mixture was worked up and the crude product was purified by automated flash chromatography (EA:Hx, 5 → 40%) affording thioindoxyl **S3** (7 mg, 29.1 µmol, 37%) as a pale yellow crystalline solid. Due to limited chemical stability of electron-rich thioindoxyls under air, the product was immediately condensed with the respective aldehyde after spectroscopic characterisation.

^{1}H-NMR ($CDCl_3$, 400 MHz): δ (ppm) = 7.05 (s, 1H), 3.97 (s, 3H), 3.95 (s, 3H), 3.86 (s, 3H), 3.75 (s, 2H); **^{13}C-NMR** ($CDCl_3$, 100 MHz): δ (ppm) = 199.3, 152.6, 148.9, 147.8, 141.7, 126.2, 103.4, 61.4, 60.8, 56.5, 39.5; **R_f** = 0.36 (blue fluorescence under illumination with 366 nm) on EA:Hx, 1:4.

2-(4-Hydroxy-3,5-dimethylbenzylidene)-5,6,7-trimethoxybenzo[*b*]thiophen-3(2*H*)-one (HITub-3)

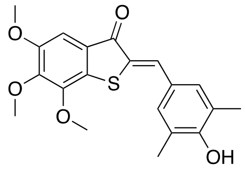

By standard procedure D, thioindoxyl **S3** (5 mg, 20.8 µmol, 1.00 equiv) was reacted with commercially available 3,5-dimethyl-4-hydroxybenzaldehyde (4 mg, 27.1 µmol, 1.30 equiv) in

dry toluene (3 mL) for 2.5 h at 80 °C then 1 h at 25 °C. Automated flash chromatography (EA:Hx, 5 → 40%) yielded **HITub-3** as an orange solid (3.4 mg, 9.1 µmol, 44%).

^{1}H-NMR ($CDCl_3$, 400 MHz): δ (ppm) = 7.84 (s, 1H), 7.38 (s, 2H), 7.23 (s, 1H), 5.08 (br. s, 1H), 4.01 (s, 3H), 3.98 (s, 3H), 3.90 (s, 3H), 2.30 (s, 6H); **^{13}C-NMR** ($CDCl_3$, 100 MHz): δ (ppm) = 188.1, 154.7, 153.0, 148.1, 147.6, 134.6, 133.0, 132.3, 128.0, 126.7, 126.2, 124.0, 104.4, 61.4, 61.2, 56.5, 16.1; **R_f** = 0.27 on EA:Hx, 1:4; ESI^+ **HRMS** for $C_{21}H_{23}O_5S^+$: calcd. m/z 373.11042, found m/z 373.11008; ESI^- **HRMS** for $C_{21}H_{22}O_5S^-$: calcd. m/z 371.09587, found m/z 371.09597.

Synthesis of HITub-4

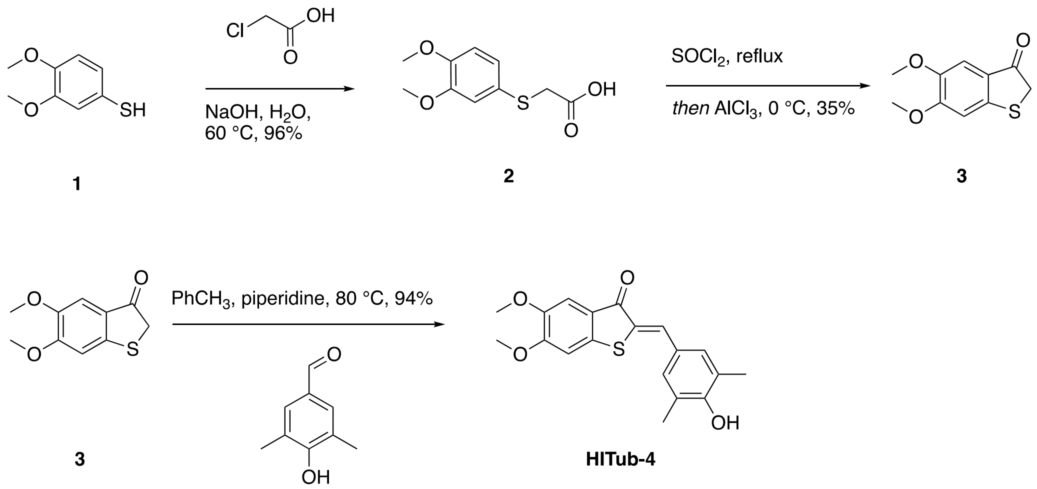

Scheme S2: Synthesis of **HITub-4**.

2-((3,4-Dimethoxyphenyl)thio)acetic acid (2)

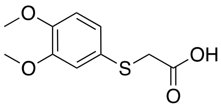

Acetic acid derivative **2** was prepared starting from commercially available thiol **1** following our previously published procedure; spectral data are in good agreement with our previously published data[3].

^{1}H-NMR (DMSO-d_6, 400 MHz): δ (ppm) = 12.67 (s, 1H), 7.00 (d, J = 1.7 Hz, 1H), 6.94 (dd, J = 1.7 & 8.3 Hz, 1H), 6.90 (d, J = 8.3 Hz, 1H), 3.74 (s, 3H), 3.73 (s, 3H), 3.67 (s, 2H); **^{13}C-NMR** (DMSO-d_6, 100 MHz): δ (ppm) = 161.3, 139.2, 138.5, 116.2, 112.9, 104.2, 102.6, 45.9, 45.8, 27.3; ESI^- **HRMS** for $C_{10}H_{11}O_4S^-$: calcd. m/z 227.03780, found m/z 227.03844.

5,6-Dimethoxybenzo[*b*]thiophen-3(2*H*)-one (3)

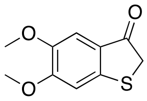

Thioindoxyl **3** was prepared following our previously reported procedure and all spectral data are in good agreement with our previously published data[3].

^{1}H-NMR ($CDCl_3$, 400 MHz): δ (ppm) = 7.17 (s, 1H), 6.82 (s, 1H), 3.94 (s, 3H), 3.87 (s, 3H), 3.79 (s, 2H); **^{13}C-NMR** ($CDCl_3$, 100 MHz): δ (ppm) = 198.6, 156.5, 149.6, 147.9, 123.7, 106.9, 105.6, 56.6, 56.3, 40.1; **R_f** = 0.33 on EA:Hx, 1:4 (blue fluorescence under illumination with 254 / 366 nm); EI **HRMS** for $C_{10}H_{10}O_3S$: calcd. m/z 210.0345, found m/z 210.1287.

2-(4-Hydroxy-3,5-dimethylbenzylidene)-5,6-dimethoxybenzo[*b*]thiophen-3(2*H*)-one (HITub-4)

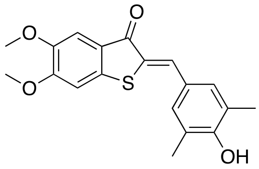

By standard procedure D, reacting thioindoxyl **3** (325 mg, 1.55 mmol, 1.00 equiv) with commercially available 3,5-dimethyl-4-hydroxybenzaldehyde (255 mg, 1.70 mmol, 1.10 equiv) in dry toluene (20 mL) for 2 h at 80 °C and overnight at room temperature. The crude product was dissolved in DMSO, water was added, the suspension was centrifuged and the supernatant was pipetted off (repeated once). The residue was dissolved in DCM, washed with water and dried over Na_2SO_4. The volatiles were removed in vacuo and the remaining solid was lyophilised to yield **HITub-4** as red solid (500 mg, 1.46 mmol, 94%).

^{1}H-NMR (DMSO-d_6, 400 MHz): δ (ppm) = 9.19 (s, 1H), 7.71 (s, 1H), 7.40 (s, 1H), 7.38 (s, 2H), 7.27 (s, 1H), 3.90 (s, 3H), 3.83 (s, 3H), 2.23 (s, 6H); **^{13}C-NMR** (DMSO-d_6, 100 MHz): δ (ppm) = 186.0, 156.2, 155.8, 148.1, 139.7, 132.9, 131.8, 127.1, 125.1, 124.9, 122.5, 107.2, 106.4, 56.4, 55.8, 16.8; **R_f** = 0.41 on EA:Hx, 1:2; ESI$^-$ **HRMS** for $C_{19}H_{17}O_4S^-$: calcd. m/z 341.08530, found m/z 341.08545.

Synthesis of HITub-2 and HITub-5

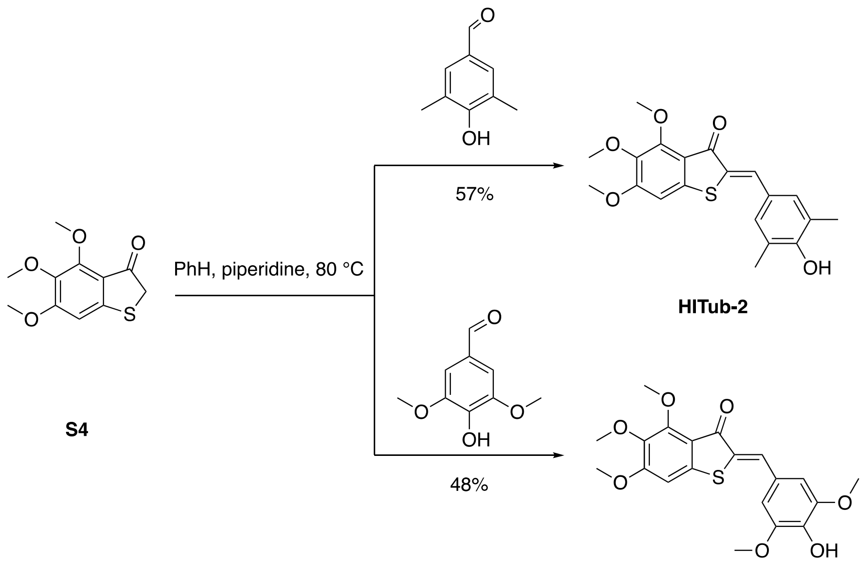

Scheme S3: Synthesis of **HITub-2** and **HITub-5**.

4,5,6-Trimethoxybenzo[*b*]thiophen-3(2*H*)-one (S4)

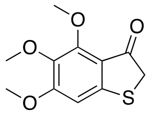

S4 was prepared following our previously reported procedure and all spectral data are in good agreement with our previously published data[3].

^{1}H-NMR ($CDCl_3$, 400 MHz): δ (ppm) = 6.59 (s, 1H), 3.97 (s, 3H), 3.89 (s, 3H), 3.79 (s, 3H), 3.74 (s, 2H); **^{13}C-NMR** ($CDCl_3$, 100 MHz): δ (ppm) = 196.5, 160.6, 154.0, 152.4, 139.3, 117.2, 101.9, 62.0, 61.5, 56.5, 40.3; **R_f** = 0.32 on EA:Hx, 1:4 (blue fluorescence under illumination with 254 nm); ESI$^+$ **HRMS** for $C_{11}H_{13}O_4S^+$: calcd. m/z 241.05291, found m/z 241.05281, for $C_{11}H_{12}O_4NaS^+$: calcd. m/z 263.03485, found m/z 263.03485.

2-(4-Hydroxy-3,5-dimethylbenzylidene)-4,5,6-trimethoxybenzo[*b*]thiophen-3(2*H*)-one (HITub-2)

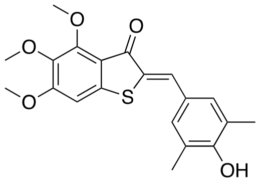

By standard procedure D, thioindoxyl **S5** (50 mg, 0.21 mmol, 1.00 equiv) was reacted with commercially available 3,5-dimethyl-4-hydroxybenzaldehyde (31 mg, 0.21 mmol, 1.00 equiv)

in dry benzene (20 mL) for 2 h at 80 °C. The crude product was dissolved in DMSO, water was added, the suspension was centrifuged and the supernatant was pipetted off (repeated once) yielding **HITub-2** as orange solid (44 mg, 0.12 mmol, 57%).

^{1}H-NMR ($CDCl_3$, 400 MHz): δ (ppm) = 7.74 (s, 1H), 7.31 (s, 2H), 6.71 (s, 1H), 5.05 (br. s, 1H), 4.05 (s, 3H), 3.94 (s, 3H), 3.85 (s, 3H), 2.29 (s, 6H); **^{13}C-NMR** ($CDCl_3$, 100 MHz): δ (ppm) = 185.8, 159.9, 154.7, 154.3, 144.0, 140.1, 132.7, 132.0, 128.3, 126.8, 123.9, 117.4, 101.7, 62.3, 61.7, 56.6, 16.1; **R_f** = 0.39 on Hx:DCM:MeOH, 1:1:0.04; ESI$^+$ **HRMS** for $C_{20}H_{21}O_5S^+$: calcd. m/z 373.11042, found m/z 373.11050, ESI$^-$ **HRMS** for $C_{20}H_{19}O_7S^-$: calcd. m/z 371.09587, found m/z 371.09634.

2-(4-Hydroxy-3,5-dimethoxybenzylidene)-4,5,6-trimethoxybenzo[*b*]thiophen-3(2*H*)-one (HITub-5)

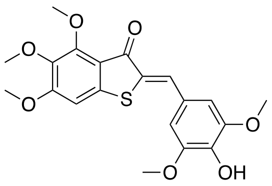

By standard procedure D, thioindoxyl **S5** (15 mg, 0.06 mmol, 1.00 equiv) was reacted with commercially available 3,5-dimethoxy-4-hydroxybenzaldehyde (14 mg, 0.07 mmol, 1.2 equiv) in dry benzene (5 mL) for 3.5 h at 80 °C. The crude was purified by flash chromatography (Hx:DCM:MeOH, 1:1:0.04) to give **HITub-5** (12 mg, 0.03 mmol, 48%).

^{1}H-NMR ($CDCl_3$, 400 MHz): δ (ppm) = 7.75 (s, 1H), 6.91 (s, 2H), 6.72 (s, 1H), 5.83 (s, 1H), 4.06 (s, 3H), 3.95 (s, 6H), 3.94 (s, 3H), 3.85 (s, 3H); **^{13}C-NMR** ($CDCl_3$, 100 MHz): δ (ppm) = 185.5, 160.1, 154.8, 147.4, 143.7, 140.2, 136.9, 132.7, 129.0, 126.2, 117.3, 108.0, 101.8, 62.3, 61.7, 56.6, 56.5; **R_f** = 0.39 on Hx:DCM:MeOH, 1:1:0.04; ESI$^+$ **HRMS** for $C_{20}H_{21}O_7S^+$: calcd. m/z 405.10080, found m/z 405.10015; ESI$^-$ **HRMS** for $C_{20}H_{19}O_7S^-$: calcd. m/z 403.08570, found m/z 403.08616.

Synthesis of HITub-1

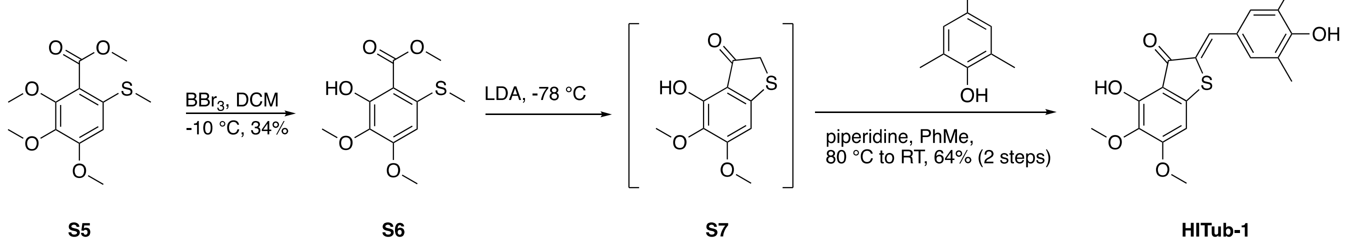

Scheme S4: Synthesis of **HITub-1**.

Methyl 2,3,4-trimethoxy-6-(methylthio)benzoate (S5)

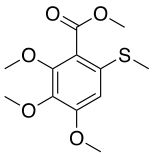

S5 was prepared following our previously reported procedure with all spectroscopical data matching the literature.[3]

Methyl 2-hydroxy-3,4-dimethoxy-6-(methylthio)benzoate (S6)

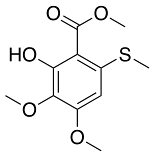

To a solution of **S5** (50 mg, 0.18 mmol, 1.00 equiv) in dry DCM (2 mL) was added BBr_3 (1 M in DCM, 0.20 mmol, 0.20 mL, 1.10 equiv) at −10 °C and the mixture was stirred for 30 min prior to quenching by the addition of an aqueous saturated solution of $NaHCO_3$. After acidification with HCl (2 M) the layers were separated and the aqueous phase was extracted with DCM. The combined organic layers were dried over Na_2SO_4 and evaporated. The crude was purified by flash chromatography (EA:Hx, 1:4) giving pure **S6** as a white solid (16 mg, 0.06 mmol, 34%).

^{1}H-NMR ($CDCl_3$, 400 MHz): δ (ppm) = 11.61 (s, 1H), 6.23 (s, 1H), 3.96 (s, 3H), 3.92 (s, 3H), 3.82 (s, 3H), 2.41 (s, 3H); **^{13}C-NMR** ($CDCl_3$, 100 MHz): δ (ppm) = 170.9, 157.8, 156.7, 139.8, 133.6, 105.0, 99.9, 60.8, 56.0, 52.1, 16.6; **R_f** = 0.34 on EA:Hx, 1:4; ESI$^+$ **HRMS** for $C_{11}H_{13}O_5S^-$: calcd. m/z 257.04892, found m/z 257.04937.

4-Hydroxy-2-(4-hydroxy-3,5-dimethylbenzylidene)-5,6-dimethoxybenzo[b]thiophen-3(2H)-one (HITub-1)

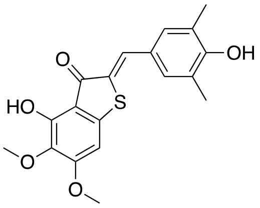

By standard procedure C, **S6** (18 mg, 0.07 mmol, 1.00 equiv) was reacted with LDA (prepared from *n*-BuLi ((2.5 M in hexane, 19 mg, 0.30 mmol, 0.12 mL, 4.33 equiv) and DIPA (32 mg, 0.32

mmol, 0.04 mL, 4.60 equiv)). After stirring for 1 h at −78 °C and 1 h at 25 °C, the mixture was worked up yielding crude **S7**.

An analytically pure sample of thioindoxyl **S7** can be obtained by purification of the crude by automated flash chromatography (EA:Hx, 5 → 100%) and was characterised as follows: **^{1}H-NMR** ($CDCl_3$, 400 MHz): δ (ppm) = 9.81 (s, 1H), 6.46 (s, 1H), 3.92 (s, 3H), 3.85 (s, 2H), 3.84 (s, 3H); **^{13}C-NMR** ($CDCl_3$, 100 MHz): δ (ppm) = 201.8, 161.7, 153.0, 150.5, 132.9, 112.4, 98.7, 61.1, 56.7, 40.5; **R_f** = 0.37 on EA:Hx, 1:4; ESI$^+$ **HRMS** for $C_{10}H_{11}O_4S^+$: calcd. m/z 227.03726, found m/z 227.03743; ESI$^-$ **HRMS** for $C_{10}H_9O_4S^-$: calcd. m/z 225.02270, found m/z 225.02256.

Due to partial decomposition during chromatography it is however more practical to use crude **S7** in the following step. By standard procedure D, crude **S7** was reacted with commercially available 3,5-dimethyl-4-hydroxybenzaldehyde (11 mg, 0.07 mmol, 1.00 equiv) in dry toluene (5 mL) for 1h at 80 °C and overnight at room temperature. After work-up, the crude product was purified by precipitation with water from a concentrated DMSO solution (repeated once) to afford **HITub-1** as an orange-brown solid (16 mg, 0.04 mmol, 64% over two steps).

^{1}H-NMR (DMSO-d_6, 400 MHz): δ (ppm) = 10.14 (s, 1H), 9.22 (s, 1H), 7.65 (s, 1H), 7.36 (s, 2H), 7.00 (s, 1H), 3.90 (s, 3H), 3.70 (s, 3H), 2.23 (s, 6H); **^{13}C-NMR** (DMSO-d_6, 100 MHz): δ (ppm) = 187.8, 170.6, 170.2, 160.5, 156.7, 152.8, 141.8, 133.8, 132.7, 132.2, 127.4, 125.6, 125.2, 111.9, 99.9, 60.8, 57.1, 17.2; **R_f** = 0.35 on DCM + 1% MeOH; **HRMS** for $C_{19}H_{19}O_5S^+$: calcd. m/z 359.09477, found m/z 359.09522; **HRMS** for $C_{19}H_{17}O_5S^-$: calcd. m/z 357.08022, found m/z 357.08054.

Synthesis of HITub-7

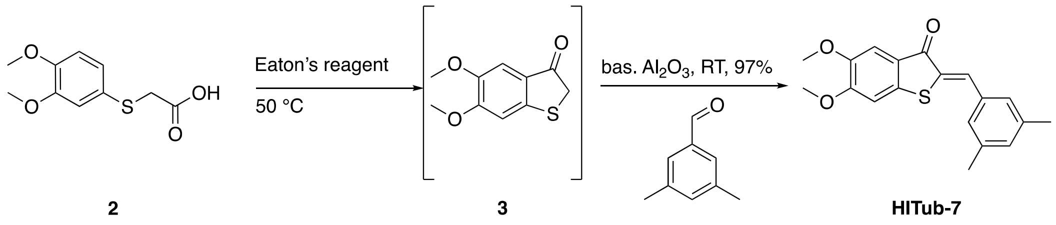

Scheme S5: Synthesis of **HITub-7**.

2-(3,5-Dimethylbenzylidene)-5,6-dimethoxybenzo[*b*]thiophen-3(2*H*)-one (HITub-7)

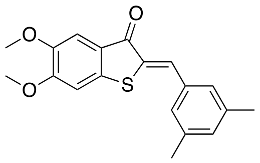

By standard procedure E, **2** (300 mg, 1.31 mmol, 4.00 equiv) was stirred with Eaton's reagent for 2 h. After extraction, commercially available 3,5-dimethylbenzaldehyde (44 mg, 0.33 mmol, 1.00 equiv) and basic Al_2O_3 were added to crude **3**. The mixture was stirred for 12 h and after work-up the crude product was purified by chromatography (EA:Hx, 1:10 → 1:4) to yield **HITub-7** (104 mg, 0.32 mmol, 97%).

^{1}H-NMR ($CDCl_3$, 400 MHz): δ (ppm) = 7.86 (s, 1H), 7.36 (s, 1H), 7.30 (s, 2H), 7.03 (s, 1H), 6.91 (s, 1H), 3.97 (s, 3H), 3.92 (s, 3H), 2.36 (s, 6H); **^{13}C-NMR** ($CDCl_3$, 100 MHz): δ (ppm) = 187.6, 156.1, 148.4, 141.2, 138.7, 134.4, 133.6, 132.1, 130.9, 128.9, 123.5, 107.9, 105.3, 56.6, 56.4, 21.5; **R_f** = 0.38 on EA:Hx, 1:2.4; EI **HRMS** for $C_{19}H_{18}O_3S$: calcd. m/z 326.4100, found m/z 326.0966.

Synthesis of HITub-6

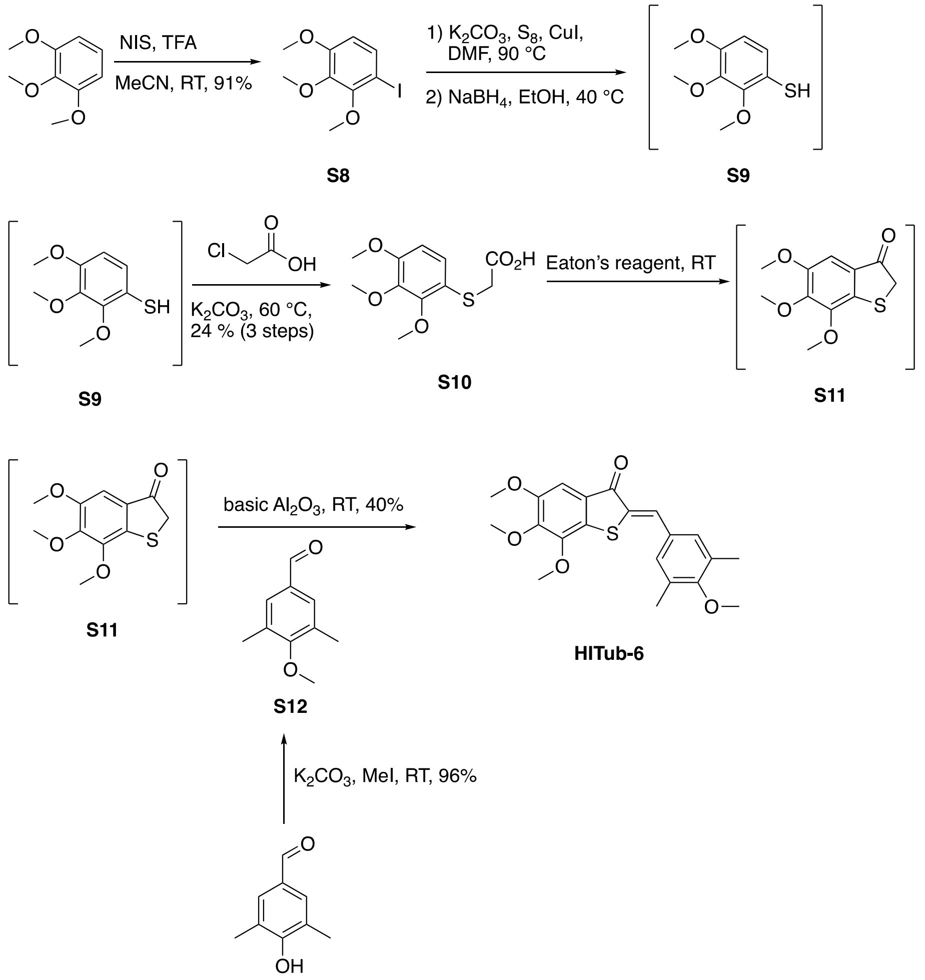

Scheme S6: Synthesis of **HITub-6**.

1-Iodo-2,3,4-trimethoxybenzene (S8)

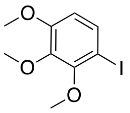

The compound was prepared following a procedure published by Castanet et al.[4] and the spectral data are in good agreement with literature.[5]

^{1}H-NMR ($CDCl_3$, 400 MHz): δ (ppm) = 7.42 (d, *J* = 8.8 Hz, 1H), 6.50 (d, *J* = 8.8 Hz, 1H), 3.88 (s, 3H), 3.86 (s, 3H), 3.87 (s, 3H) 3.85 (s, 3H); **^{13}C-NMR** ($CDCl_3$, 100 MHz): δ (ppm) = 154.5, 153.5, 142.8, 132.7, 109.9, 81.5, 61.2, 61.0, 56.3; **R_f** = 0.52 on EA:Hx, 1:5.

2-((2,3,4-Trimethoxyphenyl)thio)acetic acid (S10)

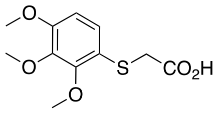

An oven-dried Schlenk tube was filled with **S8** (1.95 g, 6.61 mmol, 1.00 equiv), sulphur powder (701 mg, 21.9 mmol, 3.31 equiv), CuI (136 mg, 0.72 mmol, 0.11 equiv) and K_2CO_3 (1.80 g, 13.0 mmol, 1.97 equiv). After addition of DMF (15 mL), the tube was evacuated carefully and refilled with nitrogen. The mixture was stirred for 12 h at 90 °C. Under cooling with ice-water, ground $NaBH_4$ (891 mg, 23.6 mmol, 3.57 equiv) was added and the mixture was stirred for 5 h at 40 °C. After addition of EtOH (5 mL), the mixture was stirred for another 4 h at this temperature. Chloroacetic acid (1.50 g, 15.9 mmol, 2.41 equiv) and K_2CO_3 (0.51 g, 3.69 mmol, 0.56 equiv) were added and the reaction mixture was heated up to 60 °C for 5 h. After addition of water (5 mL), the reaction mixture was stirred for 4 d at ambient temperature. Water (400 mL) was added and it was extracted with EtOAc (3 × 50 mL). The combined organic layers were washed with $Na_2S_2O_3$ solution (1 M, 3 × 25 mL), brine (25 mL) and dried over Na_2SO_4. The solvent was evaporated under reduced pressure to yield 1.49 g of crude acid **S10**. By checking with LC–MS, desired product **S10** as well as the corresponding thiol **S9** were detected. Therefore, the alkylation procedure was repeated. The crude product was suspended in NaOH (2 M, 30 mL) and a solution of chloroacetic acid (813 mg, 8.60 mmol) in water (3 mL) was added and the reaction mixture was stirred at 60 °C for 2 h. $Na_2S_2O_3$ solution (1 M, 2 mL) was added and the mixture was stirred for another hour at 60 °C. After acidifying with concentrated HCl to pH 1, it was extracted with EtOAc (3 × 25 mL), the organic layer was washed with brine, dried over Na_2SO_4 and the solvent was removed under reduced pressure. The residue was purified by chromatography (EA:Hx:MeOH, 1:1:0 → 1:1:1) to yield **S10** as a pale yellow oil (413 mg, 1.60 mmol, 24% over 3 steps).

^{1}H-NMR ($CDCl_3$, 400 MHz): δ (ppm) = 7.14 (d, *J* = 8.7 Hz, 1H), 6.63 (d, *J* = 8.7 Hz, 1H), 3.94 (s, 3H), 3.86 (s, 3H), 3.85 (s, 3H), 3.59 (s, 2H); **^{13}C-NMR** ($CDCl_3$, 100 MHz): δ (ppm) = 175.3,

154.5, 153.6, 142.8, 128.0, 119.0, 107.8, 61.3, 61.1, 56.2, 37.0; **R$_f$** = 0.68 on EA:Hx:MeOH, 1:1:1; ESI$^+$ **HRMS** for $C_{11}H_{15}O_5S^+$: calcd. m/z 259.29545, found m/z 259.06355.

4-Methoxy-3,5-dimethylbenzaldehyde (S12)

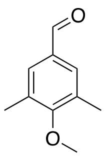

4-Hydroxy-3,5-dimethylbenzaldehyde (600 mg, 3.99 mmol, 1.00 equiv) and an excess of K_2CO_3 were suspended in dry acetone. Iodomethane (1 mL, 16.00 mmol, 4.01 equiv) was added and the reaction mixture was stirred for 12 h at ambient temperature. The solvent was removed under reduced pressure. After addition of water (20 mL), the mixture was extracted with ethyl acetate (3 × 30mL), the combined organic layers were washed with brine, dried over Na_2SO_4 and the solvent was removed under reduced pressure to yield **S12** as a colourless oil (630 mg, 3.84 mmol, 96%)

The spectral data match the literature.[5]

^{1}H-NMR ($CDCl_3$, 400 MHz): δ (ppm) = 9.87 (s, 1H), 7.55 (s, 2H), 3.77 (s, 3H), 2.34 (s, 6H); **^{13}C-NMR** ($CDCl_3$, 100 MHz): δ (ppm) = 191.8, 162.5, 132.2, 132.1, 130.8, 59.8; **R$_f$** = 0.29 on EA:Hx, 1:2.4.

5,6,7-Trimethoxy-2-(4-methoxy-3,5-dimethylbenzylidene)benzo[*b*]thiophen-3(2*H*)-one (HITub-6)

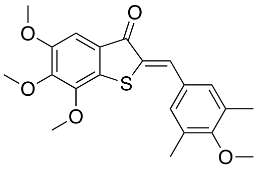

By Standard Procedure E, acid **S10** (75 mg, 0.29 mmol, 4.75 equiv) was stirred with Eaton's reagent for 1 h. After extraction, aldehyde **S12** (10.0 mg, 0. 06 mmol, 1.00 equiv) was added to crude **S11**. The mixture was stirred for 12 h. The crude product was purified by chromatography (EA:Hx, 1:10 → 1:2.4) to yield **HITub-6** as a yellow solid (8.8 mg, 0.02 mmol, 40%).

^{1}H-NMR ($CDCl_3$, 400 MHz): δ (ppm) = 7.84 (s, 1H), 7.39 (s, 2H), 7.23 (s, 1H), 4.01 (s, 3H), 3.99 (s, 3H), 3.90 (s, 3H), 3.75 (s, 3H), 2.34 (s, 6H); **^{13}C-NMR** ($CDCl_3$, 100 MHz): δ (ppm) = 188.1, 159.1, 153.1, 148.3, 147.6, 134.0, 133.1, 132.1, 132.0, 130.1, 129.5, 126.0, 104.4, 61.4, 61.2, 59.9, 56.6, 16.4; **R$_f$** = 0.36 on EA:Hx, 1:2.4; EI **HRMS** for $C_{21}H_{22}O_5S$: calcd. m/z 386.4620 m/z, found m/z 386.1187.

Part B: photocharacterisation in vitro

UV–vis spectrophotometry of bulk samples

Absorption spectra in cuvette ("UV–vis") were acquired on a Varian CaryScan 60 (1 cm pathlength). For photoisomerisation measurements, Hellma microcuvettes (108-002-10-40) taking 500 µL volume to top of optical window were used with test solution such that the vertical pathlength of the isomerisation light is less than 7 mm to the bottom of the cuvette window. Measurements were performed as stated individually. All photoisomerisations and relaxation rate measurements were performed at room temperature. "Star" LEDs (H2A1-models at 375, 450, 505, 515 and 530 nm from Roithner Lasertechnik) were used for photoisomerisations in the cuvette that were also predictive of what would be obtained in LED-illuminated cell culture. For all photoswitching studies the samples were illuminated by shining through the optical beam of the microcuvette, with additional illumination periods at the same wavelength used to verify that PSS had indeed been reached (no further evolution of the spectrum).

PSS spectra of the HITubs

HITubs show reliable and robust reversible switching in polar aprotic solvents. Samples containing 100% *Z* isomer (from incubation in the dark at 60 °C, Figures S1 and 3) are photoswitched to a PSS with a majority of *E* isomer after saturating illumination with 450 nm; subsequent illumination with 530 nm photoisomerizes back to a PSS favouring the *Z* isomer (typically almost 100% reached). We observed that **HITub-1** could be photoswitched in slightly acidified DMSO (Figure S1a) but did not show bulk photoswitching in neutral DMSO presumably due to pH-dependent fast relaxation as discussed below.

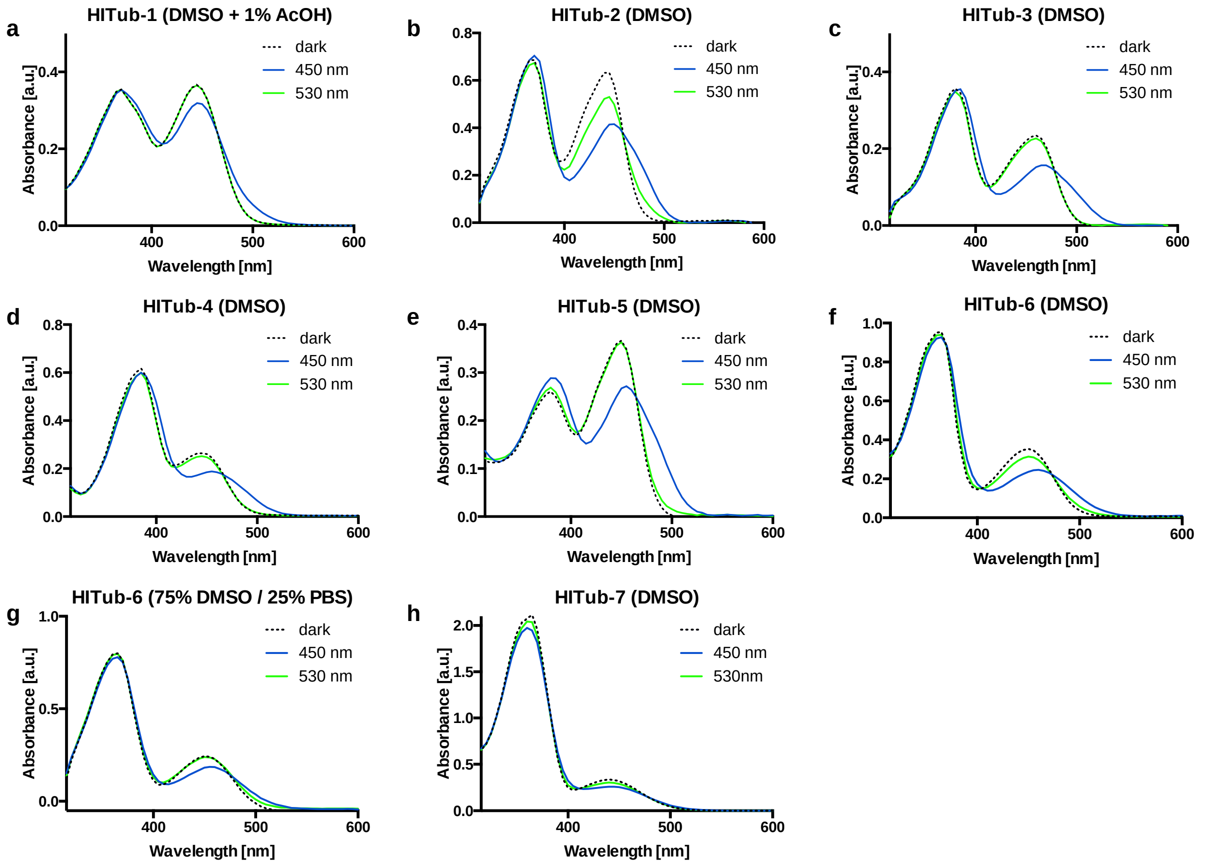

Figure S1: PSS absorption spectra of the **HITubs** at a concentration of 25 µM after incubation at 60 °C in the dark and after saturating illumination with 450 and 530 nm, respectively. All spectra in DMSO unless stated otherwise.

Solvent- and pH-dependency of photoisomerisability and relaxation rate

Addition of acid does not alter the absorption spectra or photoswitchability of **HITub-4** (Figure S2a,b). Under basic conditions however a significant hyperchromic and bathochromic shift of the absorption maximum (and to abolition of photoswitchability) is observed (Figure S2c,d). This is equally true for all **HITubs** bearing a *para*-hydroxy group on the stilbene moiety (data only shown for **HITub-4**; Figure S2c,d).We presume this results from deprotonation of the *p*-hydroxy group and resonance that substantially favours a quinoidal species, in which the photoswitchable C=C double bond of the hemithioindigo form is now tautomerised to a single bond, leading to free rotation and preventing photoswitchability. In neutral or acidic aqueous media, where the presumed quinoidal form is not present (Figure S2e-f), still no bulk photoisomerisation was observed under the applied conditions, which we presume is caused by very fast relaxation to the thermodynamically preferred *Z* isomer in aqueous media.[6]

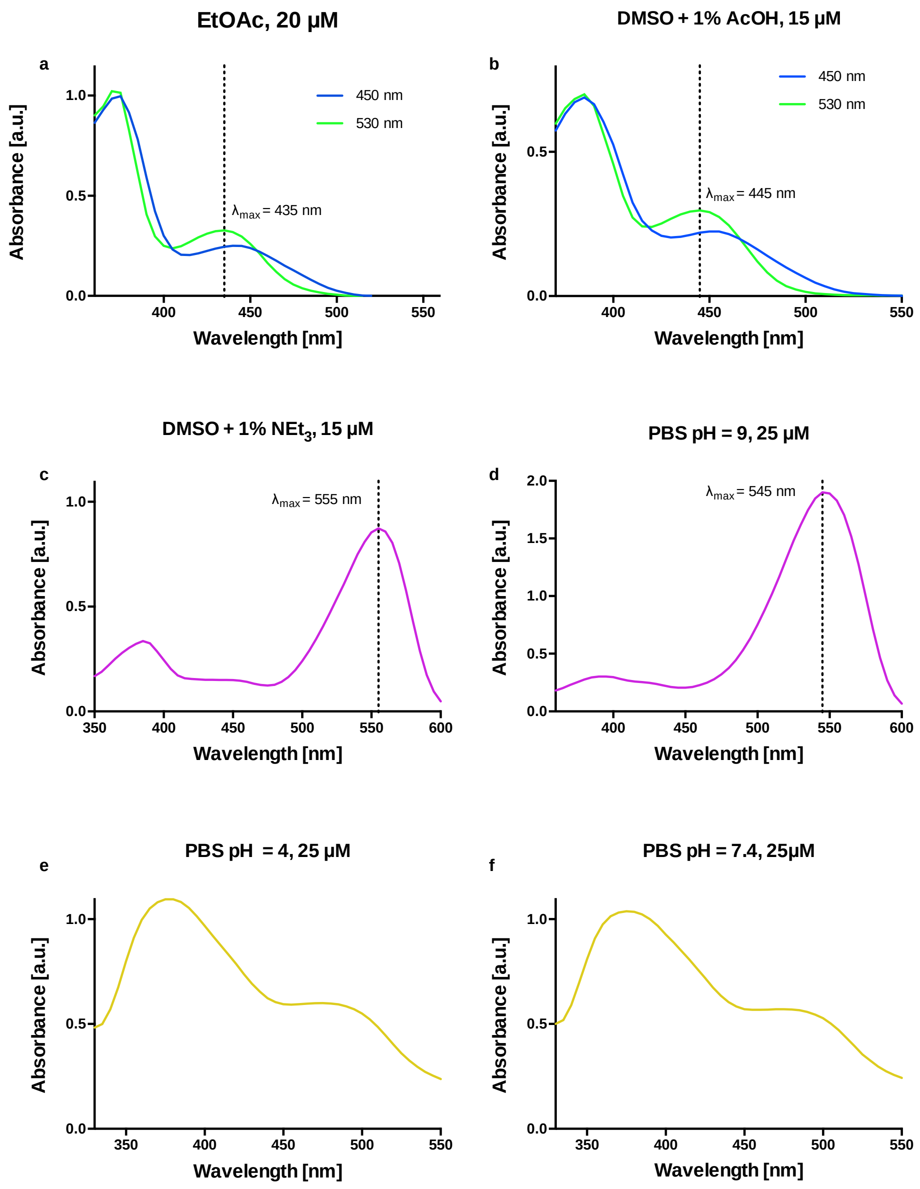

Figure S2: (**a**, **b**) Absorption spectra of **HITub-4** after saturating illumination with 450 (blue) and 530 nm (green) in neutral or acidified polar aprotic solvents, see also Fig S1d. (**c**) **HITub-4** photoswitchability could not be observed in basified aprotic media. (**d** – **f**) **HITub-4** photoswitchability could not be observed in aqueous media mixtures (75% DMSO / 25% PBS at the indicated pH).

The thermal relaxation half-life of **HITub-4** was measured at around 40 s in neutral polar aprotic solvents DMSO and EtOAc (Figure 3d and S3a) and was only slightly faster in acidified DMSO (Figure S3b). To bridge the gap towards determining a halflife in aqueous media, we measured relaxation in polar protic EtOH (Figure S3c) where the *E*-**HITub-4** thermal half-life is decreased to around 800 ms. In order to compare its performance to that of a similar *para*-hydroxy-substituted azobenzene, we attempted cuvette measurements of the thermal half-life of 4'-hydroxy-3,4,5-trimethoxyazobenzene (prepared according to literature [7]). However, neither in water nor even in DMSO could we observe that azobenzene's bulk photoisomerisation in cuvette, i.e. any spectral reversion was complete in < 12.5 ms (data not

shown) although we presume its half-life is orders of magnitude smaller as literature suggests [8]. Therefore, we concluded that the use of the HTI scaffold in this way can significantly slows spontaneous relaxation of resonance- or tautomerism-capable substituents as compared to cognate azobenzenes, and we considered there would be a chance to observe HTI isomer-dependent bioactivity in the heterogeneous cellular milieu when we applied photoswitching pulses faster than the timescale of spontaneous relaxation in aprotic media.

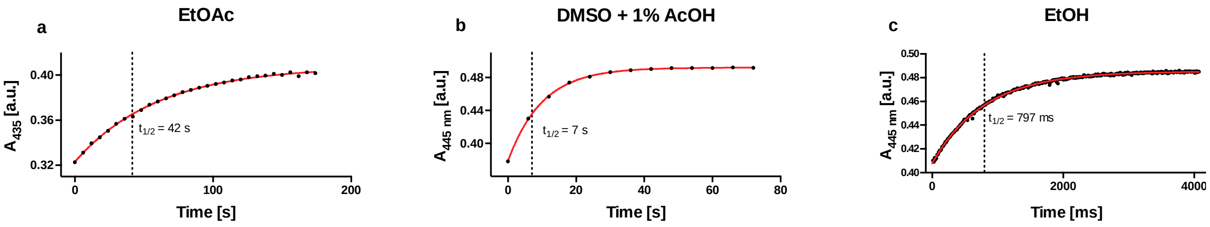

Figure S3: Thermal relaxation of **HITub-4** at 25 °C (**a**) in EtOAc, (**b**) in acidified DMSO and (**c**) in EtOH.

In these experiments, the bulk photoswitchability of *para*-hydroxy **HITub-4** was therefore confirmed in aprotic media, although not observed in protic cosolvent-aqueous media. This later seemed paradoxical when faced with its clearly photoswitchable cellular bioactivity profile. However, we note that (1) the ca. 3–4 fold biological photoswitchability index (difference between 450 nm-lit and dark-state bioactivity) of **HITub-4** and of other *para*-hydroxy **HITubs** matches closely the ca. 3.3-fold cell-free change of bioactive *Z*-isomer concentration going from dark/530 nm (100% / > 98% *Z*) to 450 nm (ca. 32% *E*) conditions, seen in PSS measurements in aprotic media; (2) these biological and concentration indices are identical to those measured for non-*para*-hydroxy **HOTubs**[3] that *are* metastable in aqueous media; (3) the solubility of **HITub-4** in pure water is very low (approx. 60 nM in distilled water, unpublished data) and it is rapidly extracted from aqueous phase into organic solvent (the limitations associated to hydrophobicity / poor solubility for further studies, such as in vivo work, are common for the vast majority of microtubule modulators of this binding site class and can be overcome by introducing water-soluble prodrug designs as we have previously shown for azocombretastatins [9]).

We theorise therefore that (a) *para*-hydroxy **HITubs** are *not* photoswitched in extracellular aqueous solution but remain as *Z* isomers there; (b) **HITubs** accumulate mainly in aprotic environments in the cell, presumably lipid membranes and vesicles, slowly exchanging across intervening (unfavoured) aqueous environments and thereby also partitioning onto hydrophobic domains of carrier protein (including binding to the colchicine site of tubulin, for active compounds); (c) under biological assay conditions the overall (*Z* + *E*) **HITub** concentration in cellular lipid environments reaches equilibrium with the extracellular applied concentration, and due to the unfavoured partitioning into water, the lipid cellular environments act as a barrier to isomer-specific equilibration of *Z*-**HITub** concentration across the membrane

between extracellular and intracellular aqueous environments; therefore (d) the *para*-hydroxy **HITub** located in aprotic environments can be photoswitched to reach its PSS there (e.g., 70:30 *E:Z* under 450 nm) while the aqueous intracellular *Z*-**HITub** concentration quickly equilibrates with this new reservoir concentration of *Z*-**HITub**. Thereby changes to the lipid reservoir *E:Z* ratio of the **HITub** (by photoequilibration) modify the intracellularly-available *Z*-**HITub** concentration that can bind tubulin, independent of the speed of *E* to *Z* relaxation in aqueous media. We have not found similar analyses in the literature of photopharmaceuticals, but we believe this principle may be found to apply in the case of other stable-isomer-active fast-relaxing photopharmaceuticals.[10]

Part C: Biochemistry and cellular biology

General cell culture: HeLa cells were maintained under standard cell culture conditions in Dulbecco's modified Eagle's medium (DMEM; PAN-Biotech: P04-035550) supplemented with 10% fetal calf serum (FCS), 100 U/mL penicillin and 100 U/mL streptomycin. Cells were grown and incubated at 37 °C in a 5% CO_2 atmosphere. Cells were typically transferred to phenol red free medium prior to assays (DMEM; PAN-Biotech: P04-03591). Compounds were applied using a minimum of co-solvent, typically 1% DMSO and were added via a D300e digital dispenser (Tecan). For assays, cells were either incubated under dark conditions (indicating that compounds were set to the all-*Z* state by thermal relaxation of the DMSO stocks at 60 °C overnight, applied while working under red-light conditions and then incubated in lightproof boxes) or lit conditions (under pulsed illumination at 450 or 530 nm using a home-made LED lighting system). "Lit" timing conditions were 75 ms pulses applied every 15 s. We controlled for unspecific phototoxicity induced by the illumination setup by including an untreated and illuminated sample in every cellular assay.

Resazurin assay: Mitochondrial diaphorase activity in HeLa cell lines was quantified by spectrophotometrically measuring the reduction of resazurin (7-hydroxy-3*H*-phenoxazin-3-one 10-oxide) to resorufin. 5,000 cells/well were seeded on 96-well microtitre plates. After 24 h, cells were treated with **HITubs**, shielded from ambient light with light-proof boxes, and exposed to the appropriate light regimes. Following 45 h of treatment, cells were incubated with 20 µL of 0.15 mg/mL resazurin per well for 3 hours at 37°C. The resorufin fluorescence (excitation 544 nm, emission 590 nm) was measured using a FLUOstar Omega microplate reader (BMG Labtech). Results are represented as percent of untreated control (reading zero was assumed to correspond to zero viable cells) and represented as mean of at least three independent experiments with SD.

Cell cycle analysis: Following 24 h of treatment with **HITubs** under the indicated light regime, cells were harvested and fixed in ice-cold 70% ethanol and incubated in a staining solution [2% DNase-free RNase A 10 mg/mL, 0.1 % Triton X-100 and 2 mg/mL propidium iodide (PI)]

for 30 min at 37 °C. Following the PI staining, cells were analyzed by flow cytometry using an LSR Fortessa (Becton Dickinson) run by BD FACSDiva 8.0.1 software. The cell cycle analysis was subsequently performed using FlowJo-V10 software (Tree Star Inc.). Results from one representative experiment are shown as a histogram. Quantification from gating on the respective histograms is shown as percent of live/singlet/PI-positive parent population per cell cycle phase across different concentrations of the compound. Every experiment was performed independently and in technical triplicates at least three times with a mean of 14,000 PI-positive singlet cells analyzed per replicate.

Resazurin antiproliferation assays

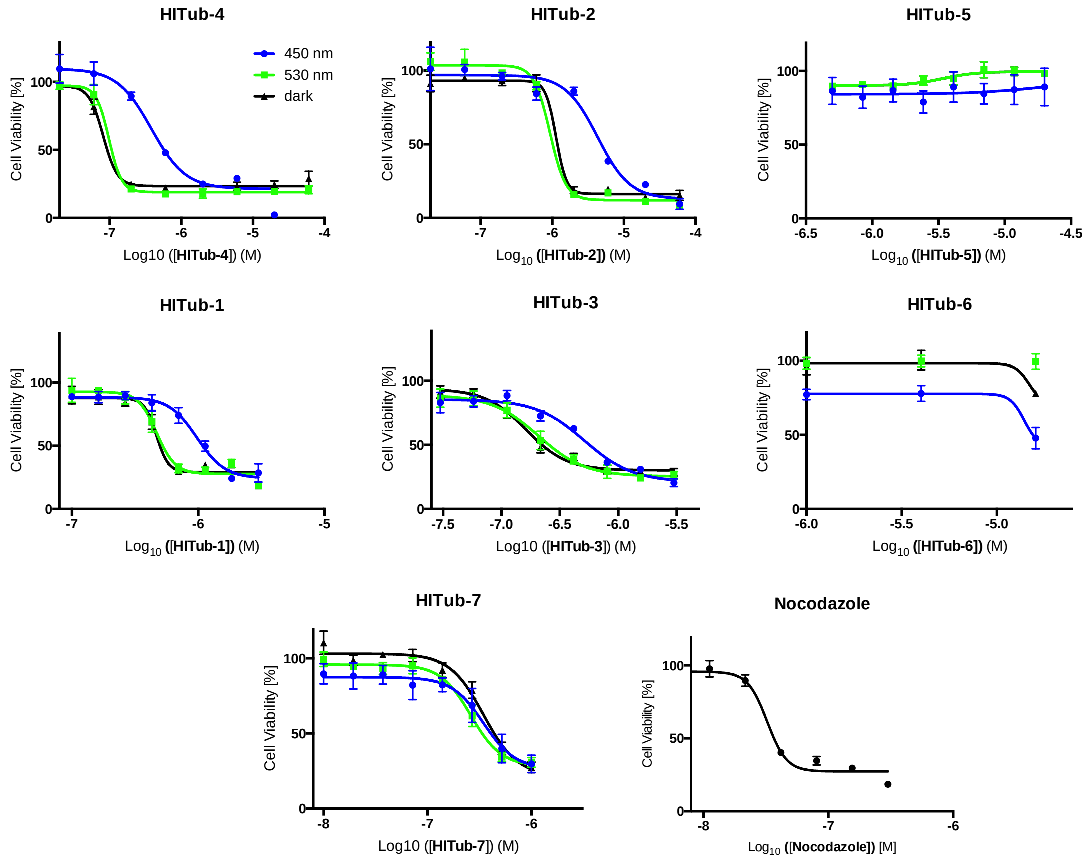

Figure S4: Representative results from Resazurin assays for **HITubs**. HeLa cells were treated with the compounds for 48 h under dark or lit (530 nm, 450 nm) conditions. Nocodazole was used as a positive reference to show antiproliferative effect in cells via microtubule depolymerisation leading towards cell death.

In vitro tubulin polymerisation assay

A **HITub-4** DMSO stock was diluted using 10% DMSO in BRB80 to reach 0.5 mM. This was mixed 1:1 with 100 mM GTP solution (in water) at room temperature. 4 µL of this mixture were spiked into ice-cold MAP-rich tubulin in BRB80 buffer (96 µL, 2 mg/mL). The sample was transferred to a quartz microcuvette held at 37 ºC in a UV–vis spectrophotometer, and was then monitored for absorbance (= turbidity) changes due to polymerisation over 20 min. A Cary 60 UV–vis spectrophotometer (Agilent Technologies) run by Cary WinUV software (version 5.0.0.999, Agilent Technologies) was used with temperature control achieved by a Cary single

cell Peltier accessory (Agilent Technologies). Absorbance data were acquired every 15 s. Generated data were processed and visualised using GraphPad Prism 6 software. Curve heights were adjusted to 0 at time zero, to account for differences in sample preparation. Since **HITub-4** cannot be isomerised in this monophasic cell-free solvent system, we did not expect to be able to observe a difference between **HITub-4** antipolymerisation activity in the dark or after 450 nm illumination, and indeed this was shown experimentally; therefore, only dark data (indicating strong polymerisation inhibition) are shown (Figure S5). We left it to cellular assays (complex multiphasic environment) to test isomer-dependent differences in bioactivity.

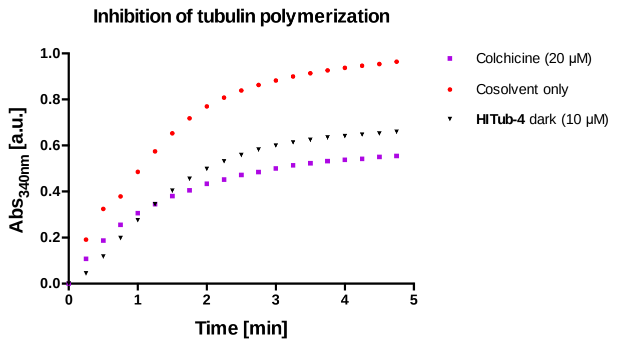

Figure S5: **HITub-4** shows *in vitro* inhibition of tubulin polymerisation (black triangle). Turbidimetric *in vitro* polymerisation assay; greater absorbance corresponds to a greater degree of polymerisation.

Immunofluorescence imaging of microtubule network structure

For fixed cell imaging (Figure 5, Figure S6), HeLa cells were seeded directly on glass coverslips in 24-well plates and treated with **HITubs**, either in the dark (all-*Z*) or under illumination by the LED array system using 450 nm LEDs (max. 70% *E*) pulsed at 75 ms "on" every 15 s, for 24 h. Then the coverslips were washed with extraction buffer (80 mM PIPES pH 6.8; 1 mM $MgCl_2$, 5 mM ethylene glycol tetra-acetic acid (EGTA) dipotassium salt and 0.5% Triton X-100) for 30 s at 37°C to remove monomeric and dimeric tubulin subunits and fixed for 10 min with a final concentration of 0.5% glutaraldehyde or else 6 min in ice-cold methanol; the glutaraldehyde-fixed cells were then quenched for 7 min with 0.1% $NaBH_4$ in PBS. Coverslips were washed with PBS twice and then blocked with PBS containing 10% FCS and 0.3% Tween-20 for 30 min at room temperature. For immunofluorescence staining of microtubules, the cells were treated with primary antibody (rabbit anti-alpha-tubulin; abcam ab18251; 1:400 in blocking buffer) for 1 h and then washed with PBS before application of the secondary antibody (donkey anti-rabbit Alexa Fluor 488; abcam ab150073; 1:400 in blocking buffer) for 1 h. The coverslips were mounted on microscopy glass slides using Roti®-Mount FluorCare DAPI (Carl Roth) and imaged with a Zeiss LSM 510, LSM 710 or SP5 confocal microscope. Image processing was performed using FIJI image analysis platform[11].

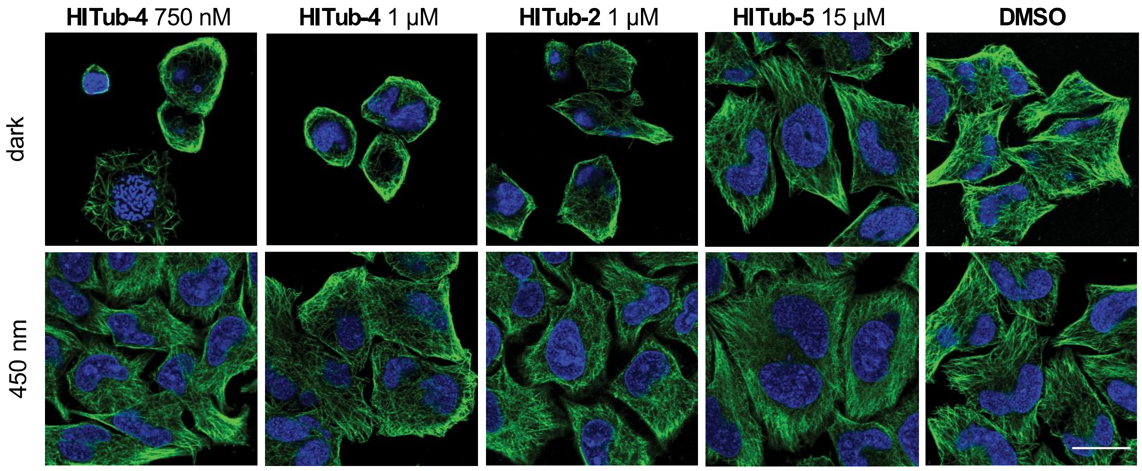

Figure S6: Immunofluorescence imaging of cells treated under lit/dark conditions with **HITub-4**, **HITub-2** and **HITub-5**, or the corresponding DMSO cosolvent controls. Microtubules of HeLa cells were immunostained with anti-α-tubulin (green), nuclei stained with DAPI (blue), scale bar = 20 µm; some images were shown in Figure **5**.

Cell cycle analysis

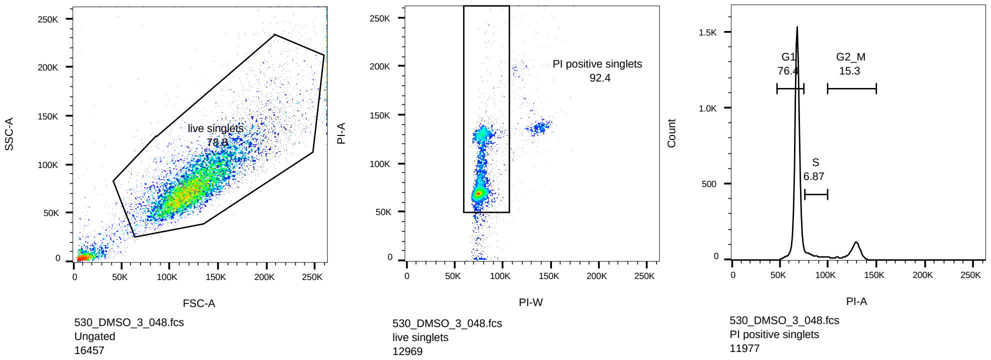

Figure S7: Representative gating strategy applied to all datasets from flow cytometric cell cycle analysis. Live singlets were determined from SSC/FSC plot, PI-positive singlets (mean of 14,000 per replicate) were then plotted as histogram and cell cycle phase gates were set to accommodate for minimal peak shifts across all samples.

Supplemental references

1. Gottlieb, H. E.; Kotlyar, V.; Nudelman, A. *J. Org. Chem.* **1997**, *62* (21), 7512-7515.
2. Suresh Kumar, A.; Thulasiram, B.; Bala Laxmi, S.; Rawat, V. S.; Sreedhar, B. *Tetrahedron* **2014**, *70* (36), 6059-6067.
3. Sailer, A.; Ermer, F.; Kraus, Y.; Lutter, F. H.; Donau, C.; Bremerich, M.; Ahlfeld, J.; Thorn-Seshold, O. *Chembiochem* **2019**, *20* (10), 1305-1314.
4. Castanet, A.-S.; Colobert, F.; Broutin, P.-E. *Tetrahedron Lett.* **2002**, *43* (29), 5047-5048.
5. Orito, K.; Hatakeyama, T.; Takeo, M.; Suginome, H. *Synthesis* **1995**, *1995* (10), 1273-1277.
6. Kink, F.; Collado, M. P.; Wiedbrauk, S.; Mayer, P.; Dube, H. *Chemistry* **2017**, *23* (26), 6237-6243.
7. Sutariya, P. G.; Modi, N. R.; Pandya, A.; Rana, V. A.; Menon, S. K. *RSC Advances* **2013**, *3* (13).
8. Garcia-Amorós, J.; Sánchez-Ferrer, A.; Massad, W. A.; Nonell, S.; Velasco, D. *Phys. Chem. Chem. Phys.* **2010**, *12* (40), 13238-13242, 10.1039/C004340K.
9. Borowiak, M.; Nahaboo, W.; Reynders, M.; Nekolla, K.; Jalinot, P.; Hasserodt, J.; Rehberg, M.; Delattre, M.; Zahler, S.; Vollmar, A.; Trauner, D.; Thorn-Seshold, O. *Cell* **2015**, *162* (2), 403-411.
10. Eisel, B.; Hartrampf, F. W. W.; Meier, T.; Trauner, D. *FEBS Lett* **2018**, *592* (3), 343-355.
11. Schindelin, J.; Arganda-Carreras, I.; Frise, E.; Kaynig, V.; Longair, M.; Pietzsch, T.; Preibisch, S.; Rueden, C.; Saalfeld, S.; Schmid, B.; Tinevez, J. Y.; White, D. J.; Hartenstein, V.; Eliceiri, K.; Tomancak, P.; Cardona, A. *Nat Methods* **2012**, *9* (7), 676-82.

Part D: NMR spectra

5,6,7-Trimethoxybenzo[*b*]thiophen-3(2*H*)-one (S3)

^{1}H-NMR spectrum

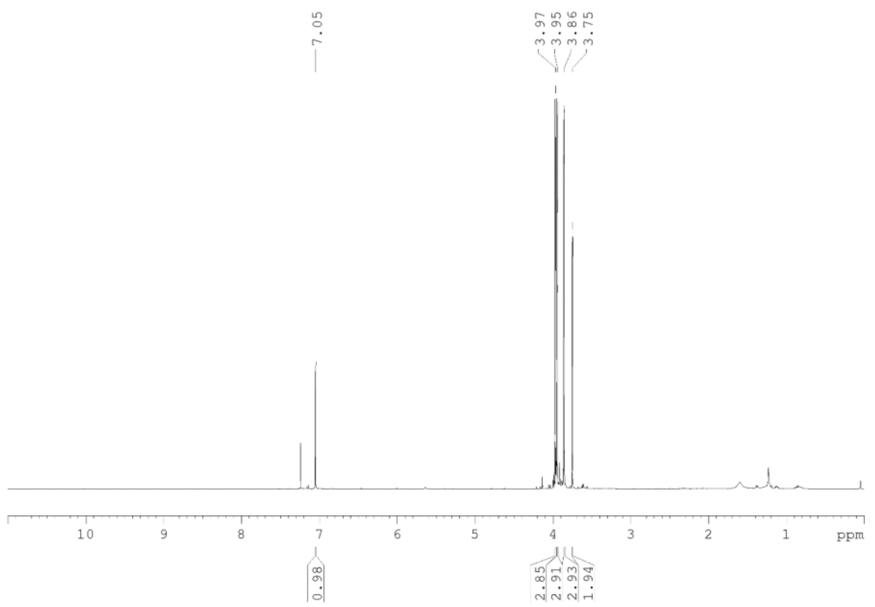

^{13}C NMR spectrum

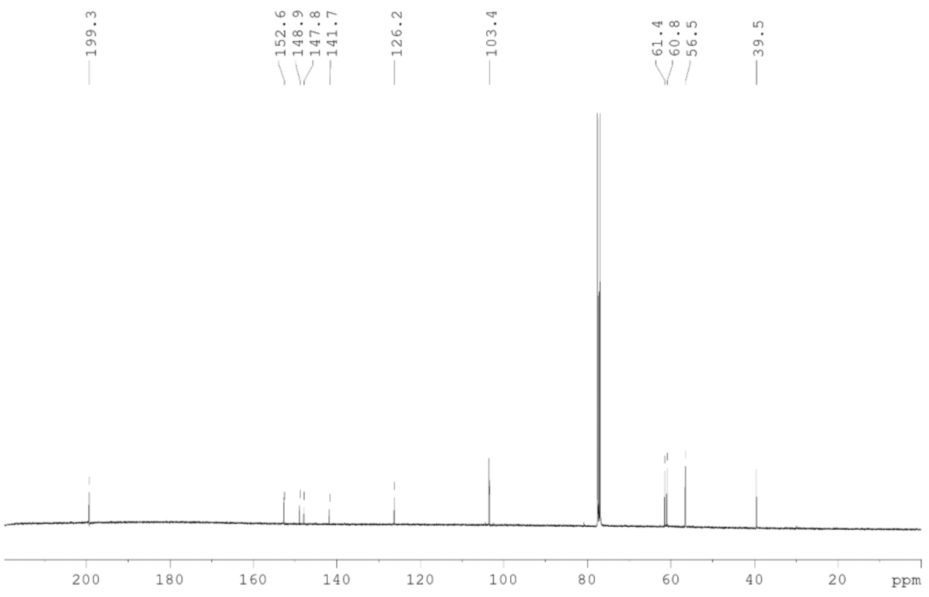

Methyl 2-hydroxy-3,4-dimethoxy-6-(methylthio)benzoate (S6)

^{1}H NMR spectrum

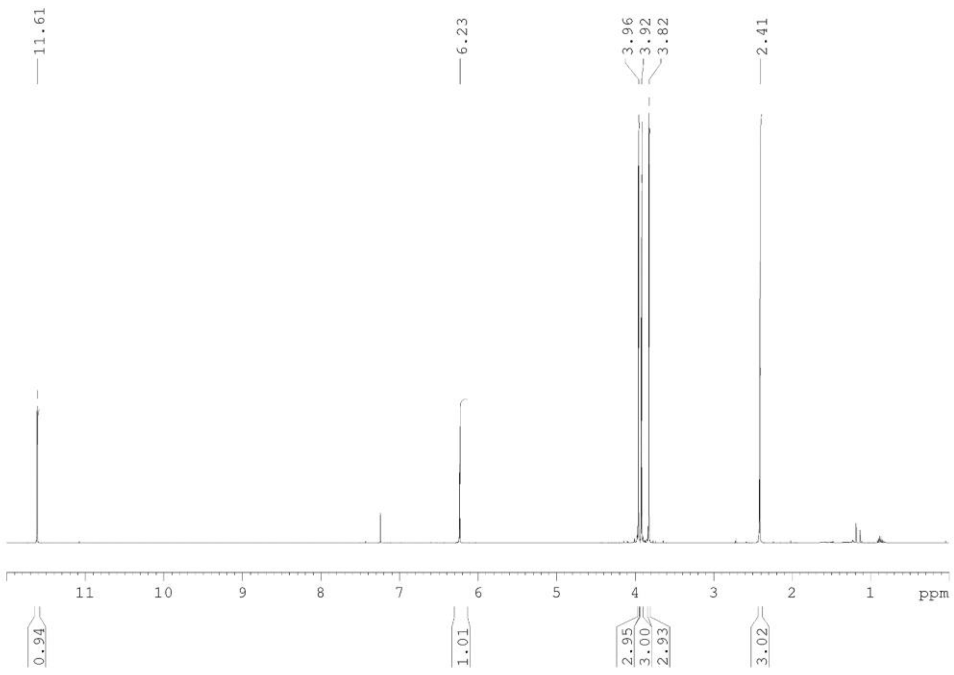

^{13}C NMR spectrum

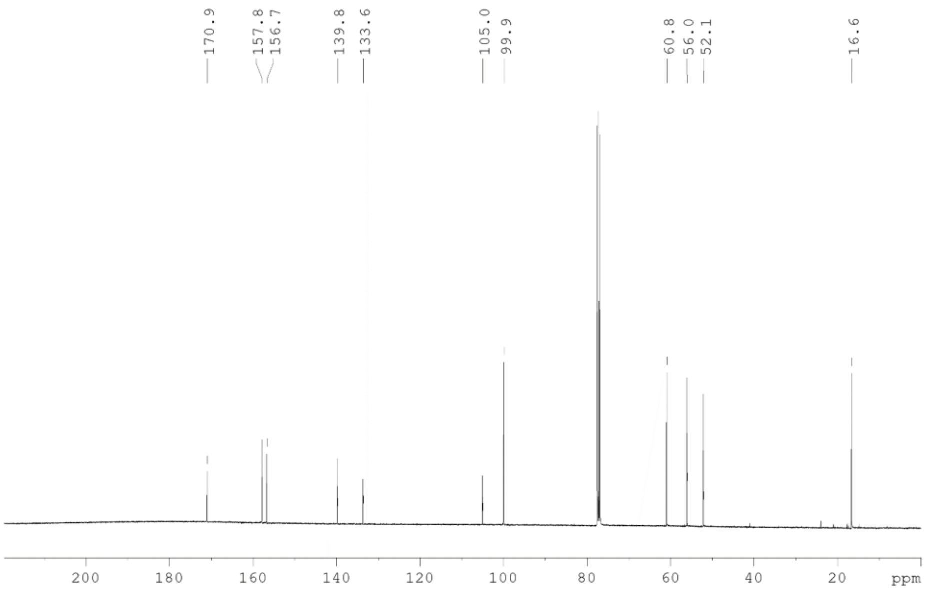

4-Hydroxy-5,6-dimethoxybenzo[*b*]thiophen-3(2*H*)-one (S7)

^{1}H NMR spectrum

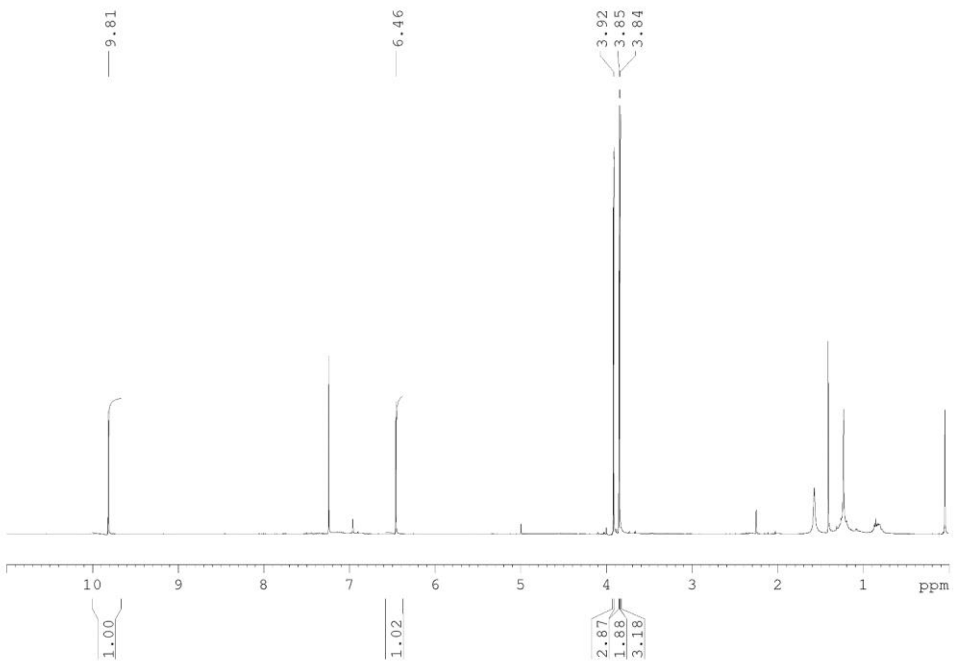

^{13}C NMR spectrum

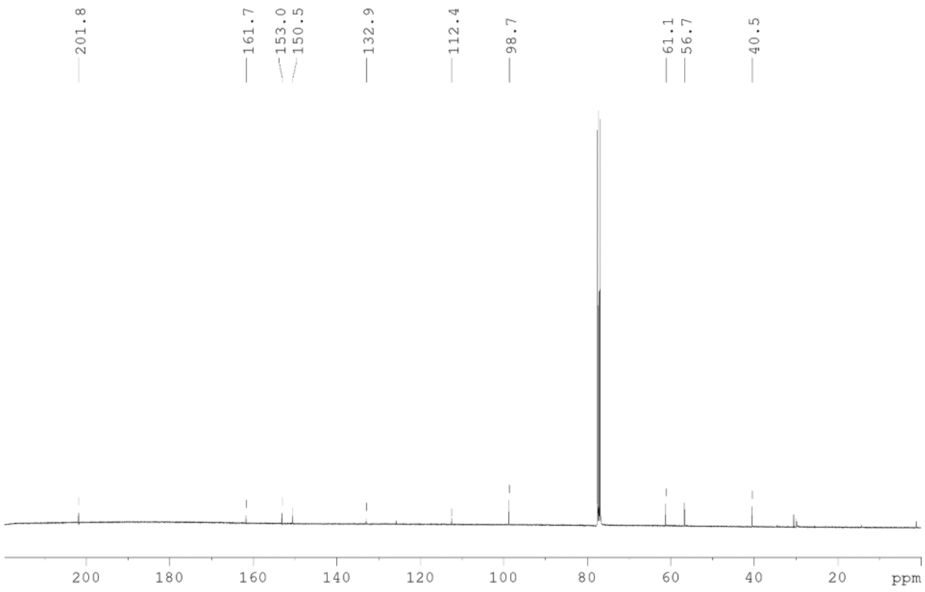

4-Hydroxy-2-(4-hydroxy-3,5-dimethylbenzylidene)-5,6-dimethoxybenzo[b]thiophen-3(2*H*)-one (HITub-1)

^{1}H NMR spectrum

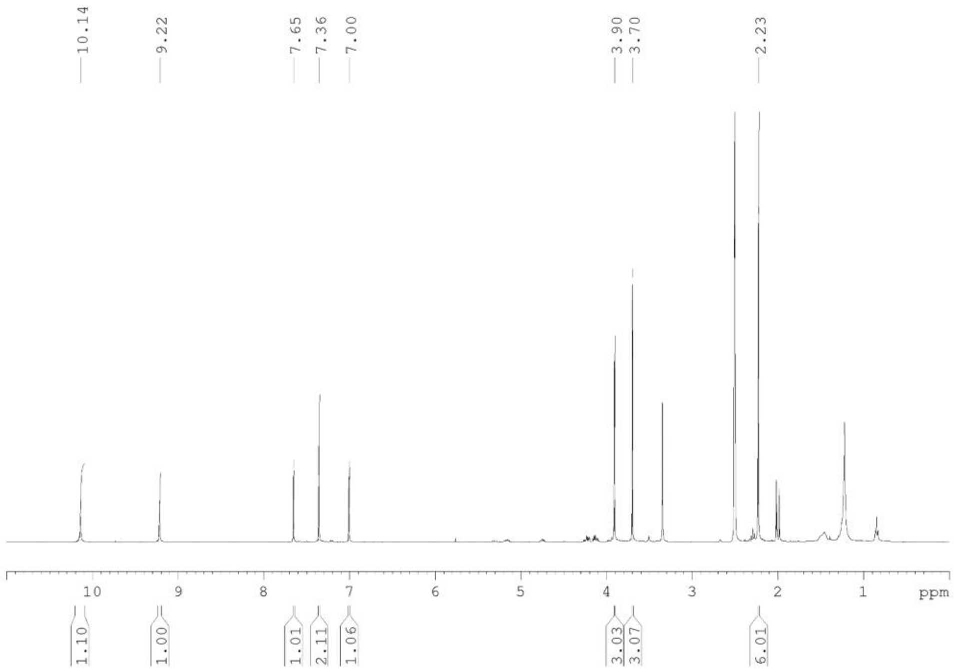

^{13}C NMR spectrum

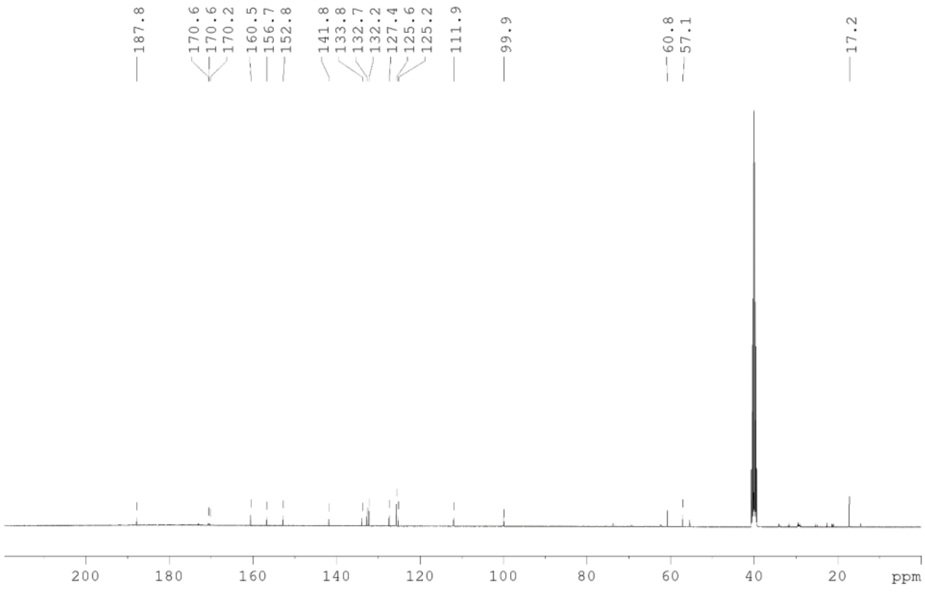

2-(4-Hydroxy-3,5-dimethylbenzylidene)-4,5,6-trimethoxybenzo[*b*]thiophen-3(2*H*)-one (HITub-2)

^{1}H NMR spectrum

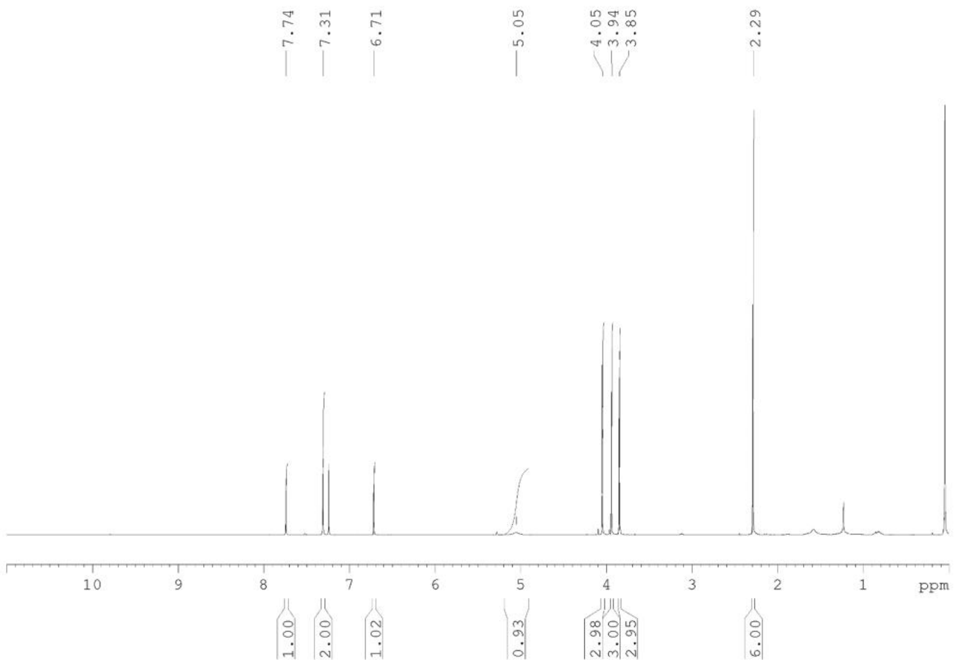

^{13}C NMR spectrum

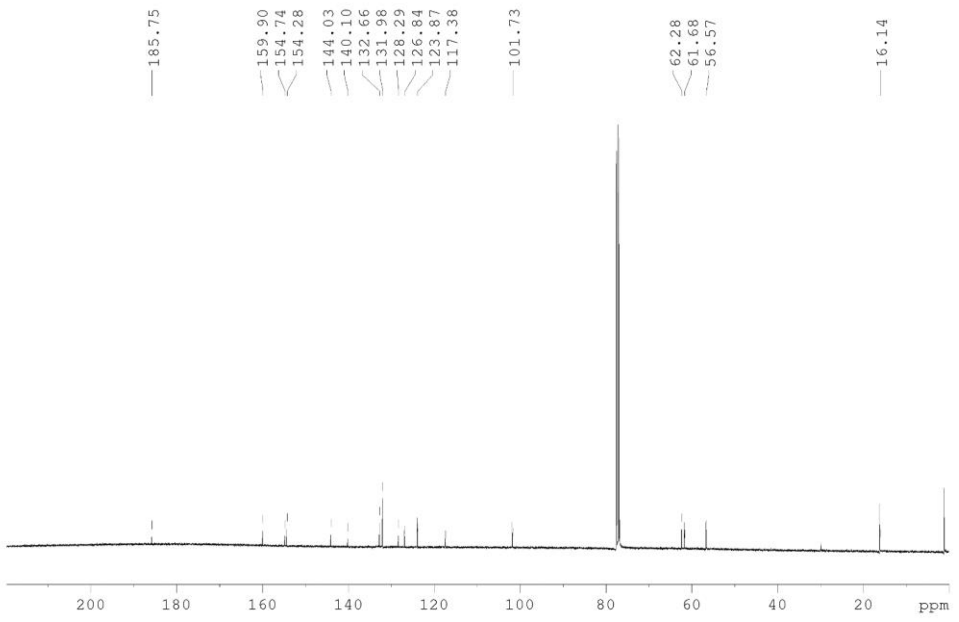

2-(4-Hydroxy-3,5-dimethylbenzylidene)-5,6,7-trimethoxybenzo[*b*]thiophen-3(2*H*)-one (HITub-3)

^{1}H NMR spectrum

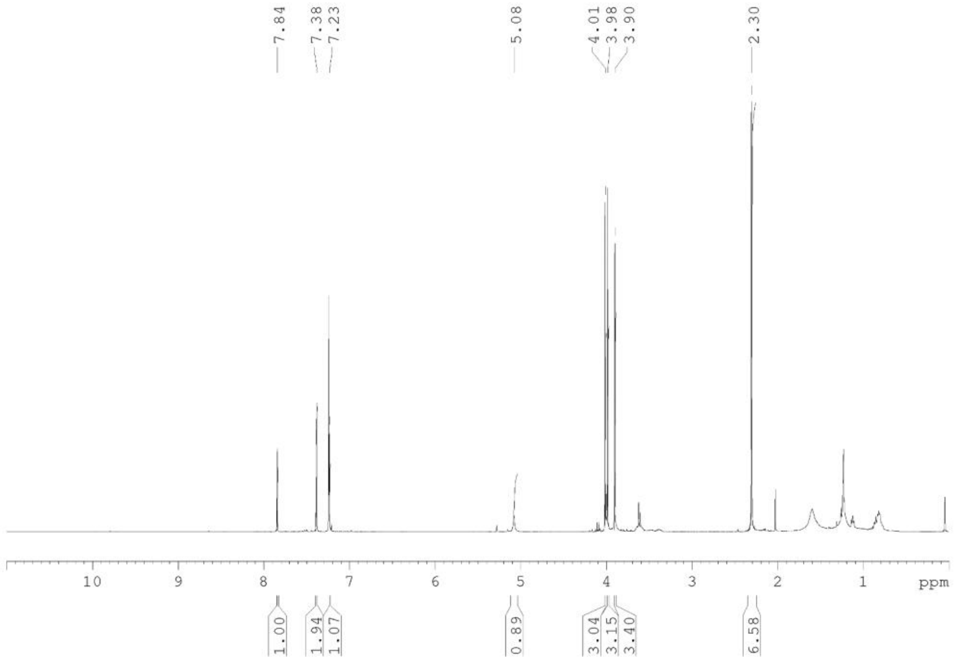

^{13}C NMR spectrum

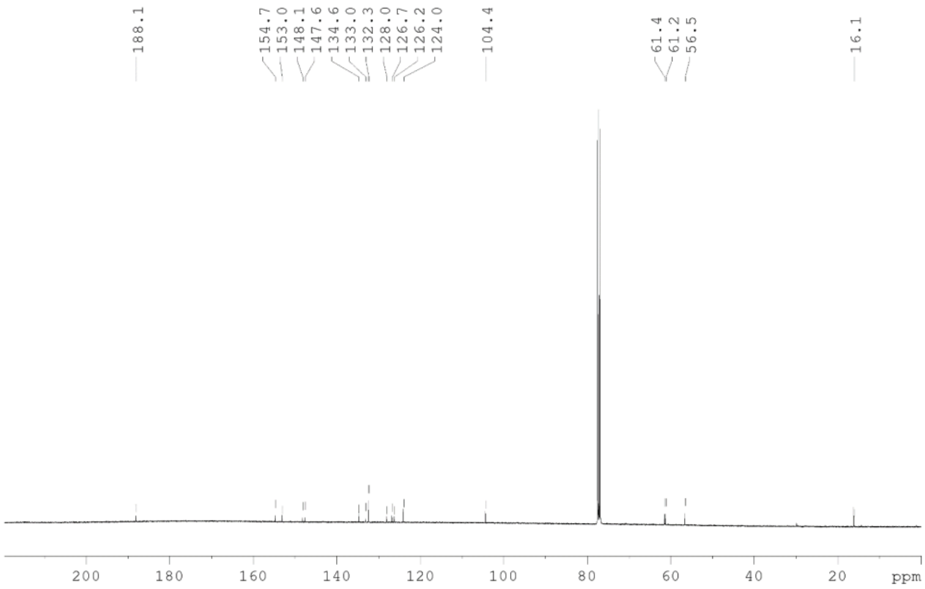

2-(4-Hydroxy-3,5-dimethylbenzylidene)-5,6-dimethoxybenzo[*b*]thiophen-3(2*H*)-one (HITub-4)

^{1}H NMR spectrum

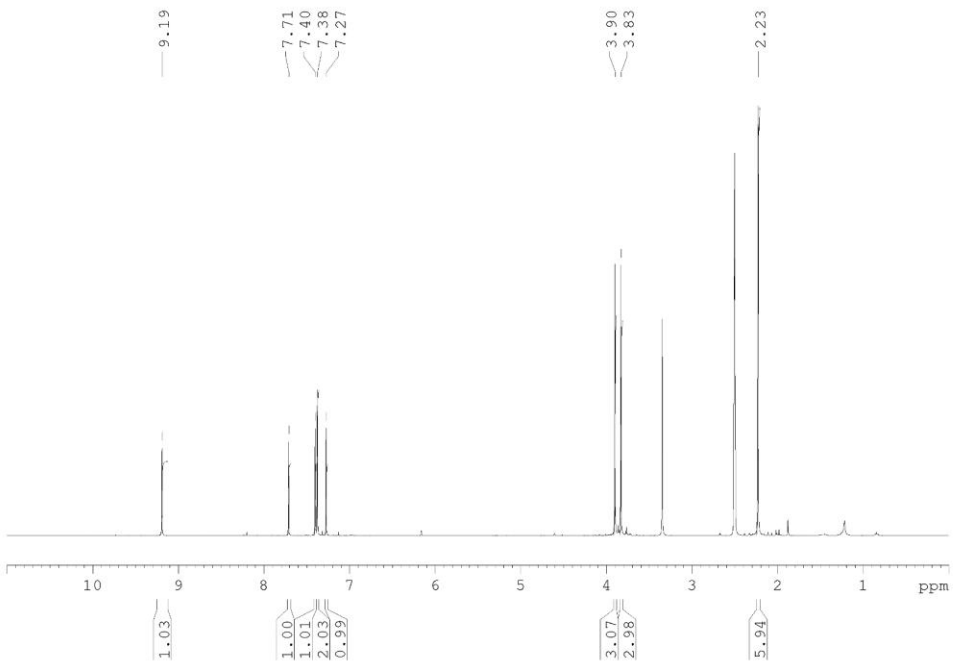

^{13}C NMR spectrum

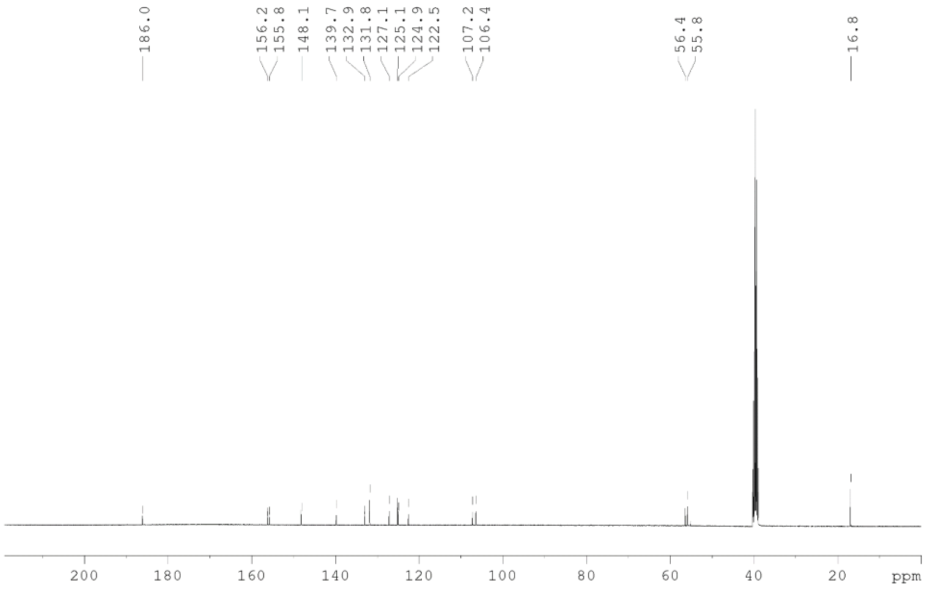

2-(4-Hydroxy-3,5-dimethoxybenzylidene)-4,5,6-trimethoxybenzo[*b*]thiophen-3(2*H*)-one (HITub-5)

^{1}H NMR spectrum

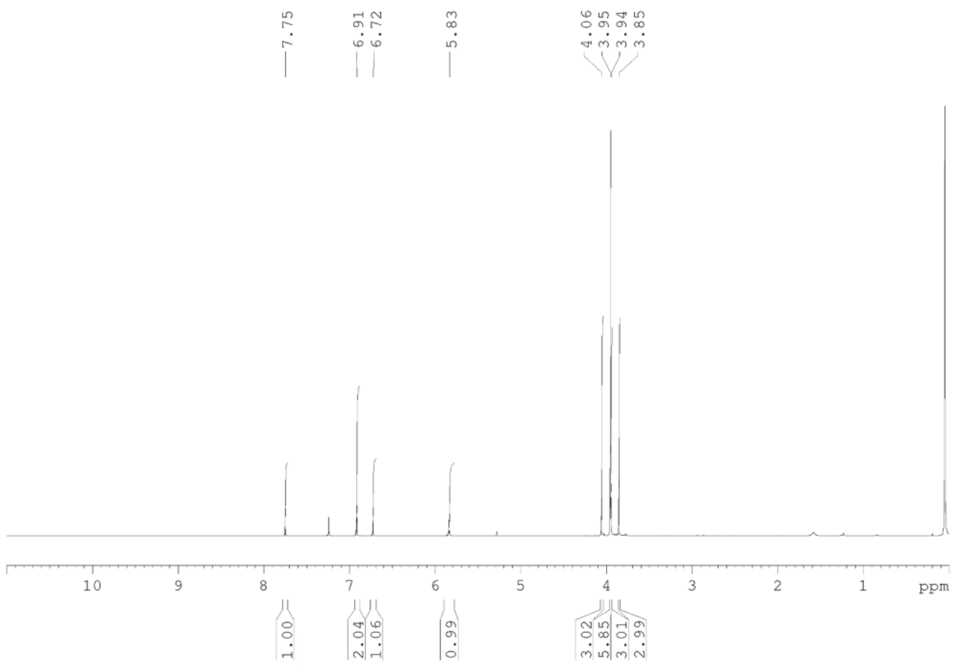

^{13}C NMR spectrum

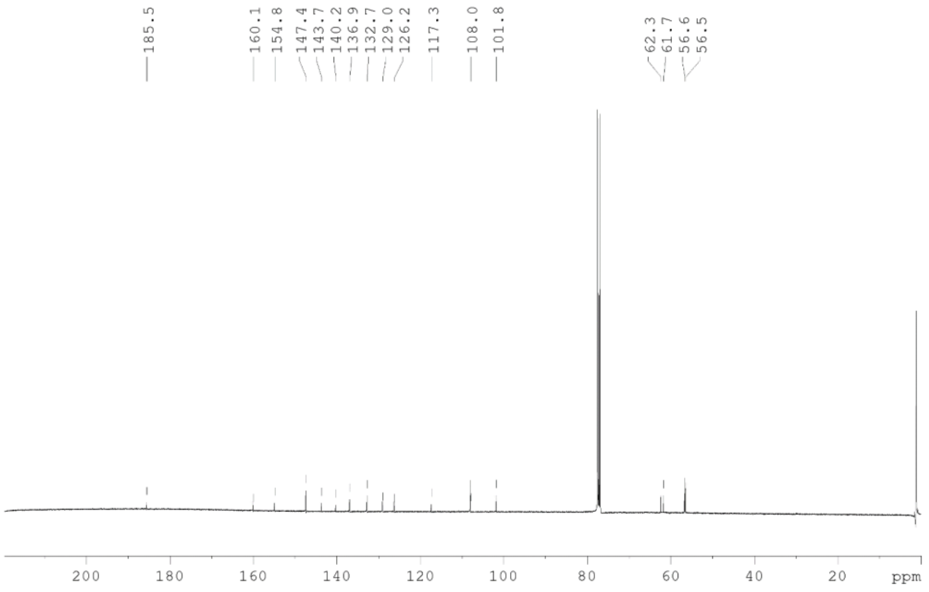

5,6,7-Trimethoxy-2-(4-methoxy-3,5-dimethylbenzylidene)benzo[*b*]thiophen-3(2*H*)-one (HITub-6)

^{1}H NMR spectrum

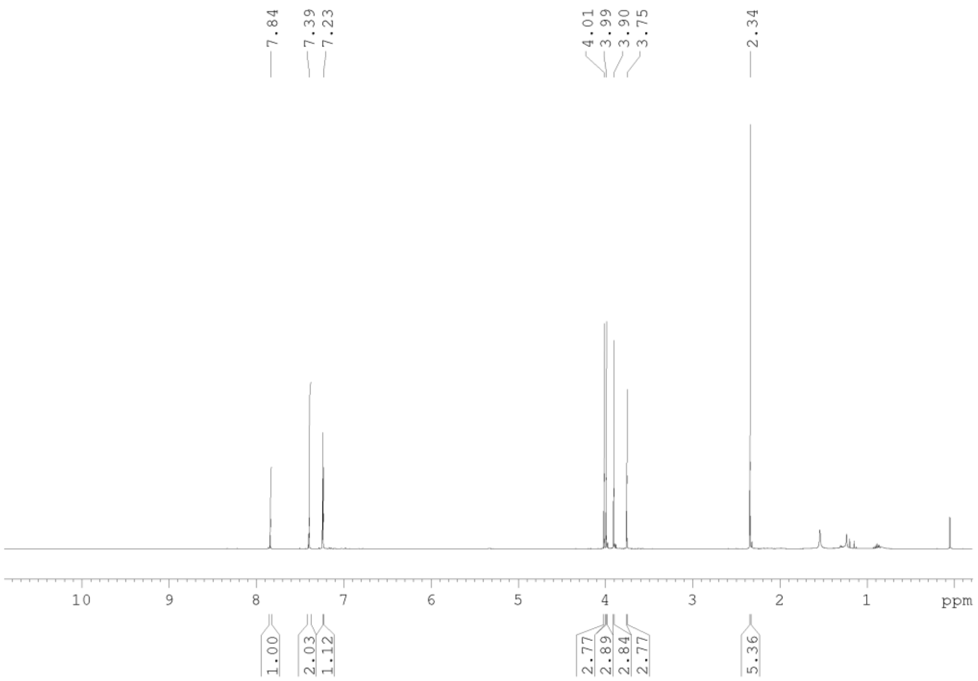

^{13}C NMR spectrum

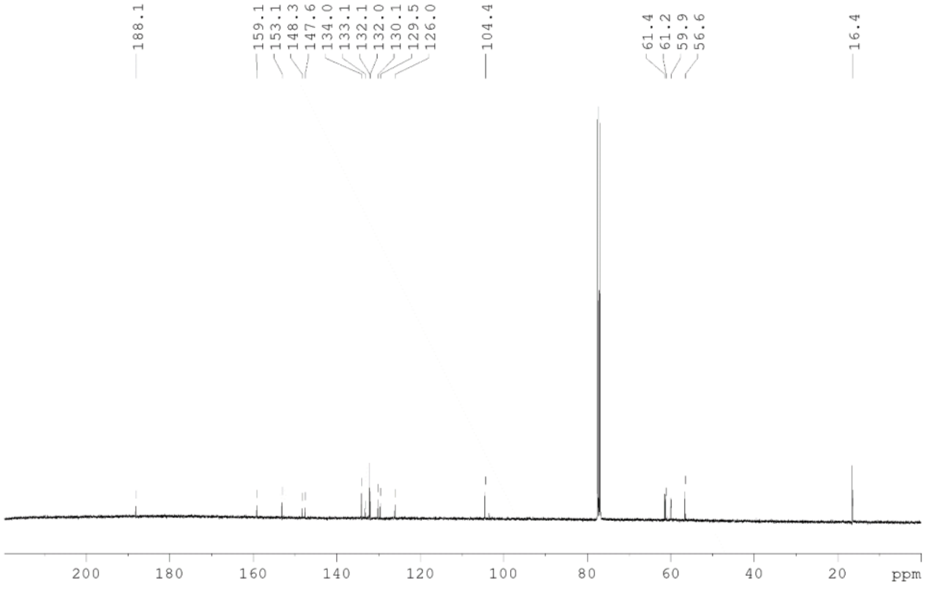

2-(3,5-Dimethylbenzylidene)-5,6-dimethoxybenzo[*b*]thiophen-3(2*H*)-one (HITub-7)

^{1}H NMR spectrum

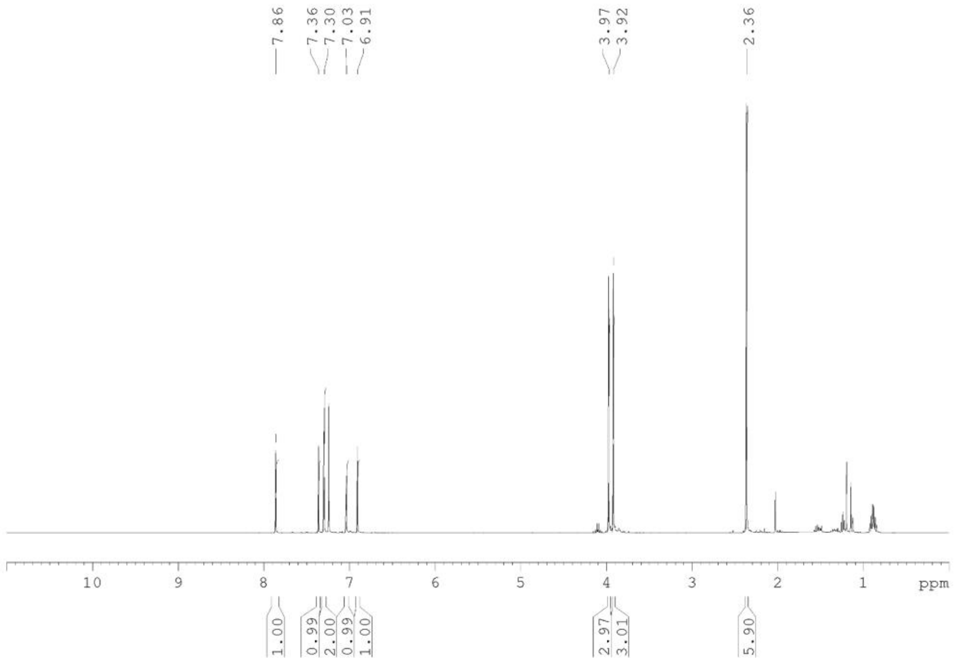

^{13}C NMR spectrum

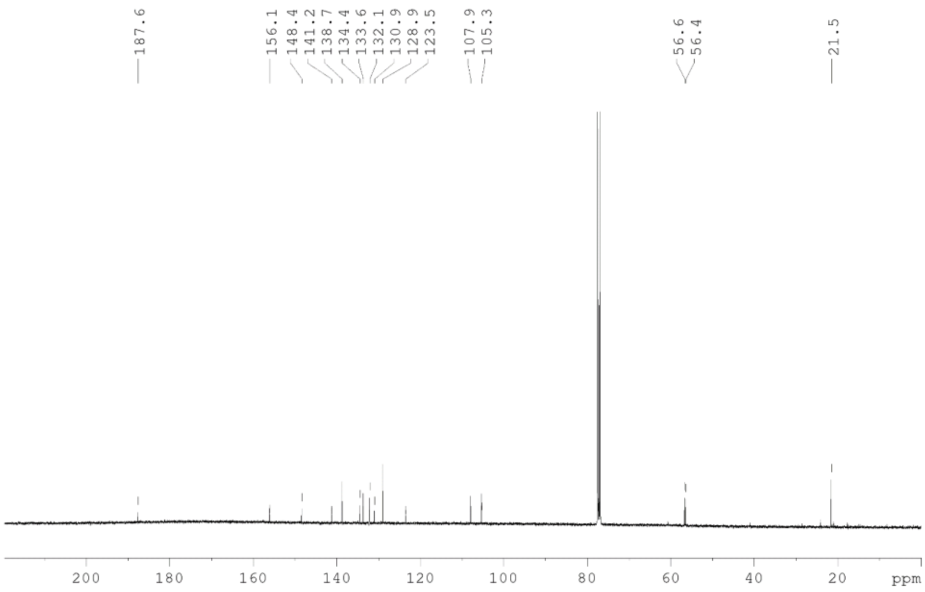

Supplement: File 1 — Full experimental protocols for chemical syntheses, photocharacterisation, biochemistry, and cell biology, including NMR spectra. [file Beilstein_J_Org_Chem-16-125-s001.pdf]
